# Genetic dissection of value-added quality traits and agronomic parameters through genome-wide association mapping in bread wheat (*T. aestivum* L.)

**DOI:** 10.3389/fpls.2024.1419227

**Published:** 2024-08-20

**Authors:** Manish K. Vishwakarma, Pradeep K. Bhati, Uttam Kumar, Ravi P. Singh, Sundeep Kumar, Velu Govindan, Gurvinder Singh Mavi, Karthikeyan Thiyagarajan, Narain Dhar, Arun K. Joshi

**Affiliations:** ^1^ Borlaug Institute for South Asia (BISA), New Delhi, India; ^2^ Astralyan Agro (OPC) Pvt. Ltd, Shamli, Uttar Pradesh, India; ^3^ International Maize and Wheat Improvement Center (CIMMYT), Texcoco, Mexico; ^4^ Indian Council of Agricultural Research (ICAR)-National Bureau of Plant Genetic Resources, New Delhi, India; ^5^ Department of Plant breeding and genetics, Punjab Agricultural University, Ludhiana, Punjab, India

**Keywords:** wheat, GWAS, quality traits, grain protein, MTAS, SNPs, end-use quality

## Abstract

Bread wheat (*T. aestivum)* is one of the world’s most widely consumed cereals. Since micronutrient deficiencies are becoming more common among people who primarily depend upon cereal-based diets, a need for better-quality wheat varieties has been felt. An association panel of 154 *T. aestivum* lines was evaluated for the following quality traits: grain appearance (GA) score, grain hardness (GH), phenol reaction (PR) score, protein percent, sodium dodecyl sulfate (SDS) sedimentation value, and test weight (TWt). In addition, the panel was also phenotyped for grain yield and related traits such as days to heading, days to maturity, plant height, and thousand kernel weight for the year 2017–18 at the Borlaug Institute for South Asia (BISA) Ludhiana and Jabalpur sites. We performed a genome-wide association analysis on this panel using 18,351 genotyping-by-sequencing (GBS) markers to find marker-trait associations for quality and grain yield-related traits. We detected 55 single nucleotide polymorphism (SNP) marker trait associations (MTAs) for quality-related traits on chromosomes 7B (10), 1A (9), 2A (8), 3B (6), 2B (5), 7A (4), and 1B (3), with 3A, 4A, and 6D, having two and the rest, 4B, 5A, 5B, and 1D, having one each. Additionally, 20 SNP MTAs were detected for yield-related traits based on a field experiment conducted in Ludhiana on 7D (4) and 4D (3) chromosomes, while 44 SNP MTAs were reported for Jabalpur on chromosomes 2D (6), 7A (5), 2A (4), and 4A (4). Utilizing these loci in marker-assisted selection will benefit from further validation studies for these loci to improve hexaploid wheat for better yield and grain quality.

## Introduction

Wheat (*Triticum* spp.) is a major staple crop in many countries, including India, and accounts for nearly 30% of global cereal consumption (van Ittersum et al., 2016). Although this main food crop is consumed chiefly as unleavened flatbread (chapati), 15% of the produced yield is used in baking for other bakery items such as bread and cookies. Wheat’s value-added characteristics are critical for home consumption and the baking industry (Cappelli and Cini, 2021). Wheat quality is currently described using a variety of metrics, and a single quality parameter can effectively distinguish wheat genotypes of variable qualities (Guzmán et al., 2019). Grain appearance, test weight, grain protein, grain hardness, sedimentation, gluten content, gluten index, iron, zinc, phenol score, and flour extraction are used to categorize wheat varieties suitable for specific end-products such as bread, biscuits, and chapatis. Wheat varieties have been divided into distinct product-specific genotypes based on these qualities in different nations.

The GlutoPeak test is used to forecast the baking qualities of wheat flour, and the association of GlutoPeak indices with several conventional quality measures such as grain hardness (GH), sodium dodecyl sulfate sedimentation value (SDSS value), farinograph, and alveograph has been investigated (Mecitoğlu Güçbilmez et al., 2019). The SDSS test is a quick test to forecast baking quality and gluten strength in wheat (Carter et al., 1999). Low alveograph stability, strength, P/L ratio, protein content, and high alveograph extensibility and biscuit diameter relate to soft endosperm genes in wheat, which are responsible for enhanced biscuit-making capacity. Ma and Baik (2018) reported that soft wheat varieties with low protein content (7.9–9.7%), low sedimentation volume (20.0–32.0 mL), and low damaged starch contents (1.9–3.4%) are desirable for good biscuit-making quality. Various physio-chemical parameters such as grain appearance (GA) score, grain hardness (GH), test weight (TWt), thousand kernel weight (TKW), protein, gluten content and index, SDSS value, phenol test, carotenoids, and diastatic activity are known to have a role in chapati-making quality (Kumar S. et al., 2018). In addition to this, for making a good loaf of bread the combination of elastic gluten with grain protein content of 13% is a prerequisite (Shuey, 1960). Wheat cultivars that have sedimentation values between 35 and 50 cc are typically used to make chapatis, while higher values than that are utilized to make bread (Mecitoğlu Güçbilmez et al., 2019). While GH and diastase activity play a clear role, it was found that phenol score may not be a good indicator of chapatti quality.

Although quality traits are important, bread wheat’s grain yield (GY) potential and stress tolerance must be increased to ensure global food security and fulfill future demands. Amid mounting breeding efforts, the low annual rate of GY increase (0.9%) (Ray et al., 2013), the growing menaces of heat and drought stresses on wheat yields (Zampieri et al., 2017), patterns of GY stagnation (Ray et al., 2012), invite the complementation of traditional breeding approaches with genomic tools that can hasten the development of high-yielding and stress-resilient wheat varieties. Wheat GY, however, has remained a challenging trait for genomic breeding due to its quantitative genetic regulation, including numerous loci with minor effects, a shortage of knowledge about the genetic basis of GY, unstable GY quantitative trait loci (QTL) reported in a different environment, epistatic effects, low heritability of GY across environments, and genotype × environment interactions (Jiang et al., 2017). Therefore, to effectively use genetic resources in breeding programs to increase wheat grain production, we must improve our knowledge of the genetic architecture of grain yield and other related attributes.

The molecular basis of complex traits is frequently studied via QTL mapping based on linkage analysis. However, mapping populations such as recombination-inbred lines (RILs) take a long time and much money to create. Furthermore, linkage mapping is based on recent recombination events, resulting in low mapping resolution, and only two alleles from the parents are considered. A genome-wide association study (GWAS) based on linkage disequilibrium (LD) represents an alternate strategy for examining connections between genotype and phenotype with the introduction of high-throughput sequencing technology (Gupta et al., 2020).

A GWAS has various advantages compared with linkage mapping, including a greater resolution and the ability to detect more variation without requiring mapping populations. GWAS has been successfully performed to explore various traits in a range of crops. In wheat, GWAS has been used to investigate grain yield, agronomic traits (Liu et al., 2017), and disease resistance (Liu et al., 2017; Riaz et al., 2018. However, only some studies have focused on quality-related traits in wheat under environmental stresses and grain yield-related traits. Hence, the main goal of this study was to use the mixed-linear model for GWAS of value-added traits and grain yield-related traits using 154 advanced breeding lines of genomic selection nurseries grown at the Ludhiana and Jabalpur Borlaug Institute for South Asia (BISA) sites. We also analyzed the phenotypic distributions of the traits and the statistical correlations between these traits. In addition, we used the KnetMiner to explore the homologous genes in other species with the reported marker trait associations (MTAs) in this study. Based on the available literature, this is the first GWAS using genotyping-by-sequencing (GBS) to examine the stability of value-added quality traits in spring wheat. Our findings provide an understanding of the genetic pathways underlying quality-related attributes.

## Materials and methods

### Plant material and phenotyping of grain yield and related traits

The panel of 154 selected advanced breeding lines of wheat (**Supplementary Table 1**) was evaluated in field trials at the BISA research farms, Jabalpur (JBP) (23^°^14′00.6N and 80^°^04′40.7E) and Ludhiana (LDH) (30^°^59′28.74N and 75^°^44′10.87E). The alpha-lattice experimental design was followed in two replications. The plot size was 5.016 m2, and the lines were sown in six rows, 22 cm apart and 3.8 m in length. The field trials were managed by standard agronomic practices recommended for the locations. Fertilizer was applied with the proportions of 150 N/60 P/40 K kg/ha at Ludhiana and 120 N/60 P/40 K kg/ha at Jabalpur as per the wheat growing zone recommendations.

During the 2017–18 crop season, the lines were phenotyped and evaluated across the location for five traits such as days to heading (DTHD) and days to maturity (DAYSMT). DTHD and DAYSMT were measured as the total number of days from sowing to when 75% of plants had either spike emergence or matured, respectively. Plant height (PH) was recorded from the plant’s base to the tip of the spike (excluding awns). Thousand kernel weight (TKW) and grain yield (GY) were measured per plot.

### Estimation of value-added quality traits

Quality traits data was recorded at the Wheat Quality Laboratory, Punjab Agricultural University (PAU), Ludhiana, Punjab for six grain quality parameters including protein percent, TWt, GA, PR score,SDSS value, and GH.

Using an Infratec 1226 Cold Grain Analyzer and the AACCI standard procedure, protein percentage was measured non-destructively at 12% moisture basis. The instrument uses near-infrared light transmitted through the grains. The results are displayed as % protein content as per calibration.

TWt, also known as hectoliter mass, measures the volume of grain per unit. Hectoliter weight was determined using a Tecator model FP Auto 680 by taking wheat grains in a 100 mL measuring cylinder; the sample was weighed, and the hectoliter weight was expressed as kg ha−1 (AACC, 2000).

Subsequently, we measured the GA score through direct visualization based on the grain’s size, shape, color, and luster. It was evaluated subjectively out of a maximum score of 10. The phenol reaction score was evaluated by soaking about 100 grains overnight in 1% phenol solution. The grains were assessed for the extent of darkness out of a score of 10, half an hour after draining off the phenol solution.

The SDSS test was used since it is a simple, small-scale method that estimates wheat gluten strength quickly. The SDSS test was carried out according to Nakamura et al., (2012). The SDS-lactic acid solution was prepared by dissolving 20 g of SDS in 1 L of distilled water and adding 20 mL of stock diluted lactic acid solution (one-part lactic acid plus eight parts distilled water volume by volume). Six grams of the whole meal sample were placed in a stoppered, graduated cylinder with 50 mL of water. The samples were mixed, hydrated for 2 min, remixed, and then hydrated for another 2 min. SDS–lactic acid solution (50 mL) was added to each sample, and the contents were mixed by inverting the tubes four times. The contents were allowed to settle, and the sedimentation height (mL) was recorded. If the value was more than 60 mL, it was considered as strong gluten wheat; from 30 to 60, it was medium strong, and if less than 30 mL, it was weak.

The GH was measured using the grain hardness tester supplied by M/S Ogawa Seiki Co. Ltd., Japan, by crushing randomly taken ten grains one by one, considering the weight, diameter, and moisture of the grain. The mean force (kg) required to crush the grain was recorded (Heo and Sherman, 2013).

### Statistical analysis

The experimental design in each environment was an alpha-lattice with two replications per environment/location. The best linear unbiased prediction (BLUP) values were obtained through META-R v6.03 (Alvarado et al., 2020). All effects are considered random for calculating the BLUP and broad-sense heritability. The correlation matrix between the BLUP values of studied traits was computed and visualized with the ‘corrplot’ package in the R software.

### Genotyping, linkage disequilibrium

Genomic DNA of the lines was isolated from 15 days-old seedling leaves using a standard cetyltrimethyl ammonium bromide (CTAB) method (Doyle and Doyle, 1987). DNA concentration was quantified using the Quant-iT PicoGreen dsDNA assay (Life Technologies Inc., NY) and normalized to 20 ng/μl. The panel of 154 lines was genotyped using the GBS method (Poland et al., 2012). The single nucleotide polymorphisms (SNPs) were called using the TASSEL (Trait Analysis by association Evolution and Linkage) version V5.3.1 GBS pipeline (Glaubitz et al., 2014). Marker polymorphisms were found using a minor allele frequency of 0.01, which resulted 13,082,477 GBS tags. Among these, 68.98% were aligned to RefSeq v1.0 using Bowtie2 (Langmead and Salzberg, 2012) with assembly of Chinese Spring (IWGSC, 2018). After filtering the tags as described by Juliana et al. (2019), we found 89,863 SNPs. Then, these markers were filtered in panel, and those with more than 60% missing data, a minor allele frequency of less than 5%, or heterozygosity of less than 10% were eliminated. Similarly, the markers and lines with a total missing data percentage of more than 50% were eliminated and 18,351 polymorphic markers were used for all the subsequent analyses. LD analysis was performed using TASSEL V5.3.1 software (Bradbury et al., 2007) using the markers with known positions from the 18,351 polymorphic markers. The LD was estimated as squared allele frequency correlations (R^2^). P-values <0.01 for each pair of loci and Bonferroni correction <0.2 were considered significant.

### Genome-wide association scans for grain quality and agronomic traits

Six grain quality parameters (GA Score, GH, PR Score, Protein %, SDSS Value, and TWt), and five agronomic traits (DTHD, DAYSMT, PH, GRYLD, and TKW) from both the locations (Ludhiana and Jabalpur) were considered for a GWAS using 18351 polymorphic GBS markers. GWAS analysis was performed with TASSEL V5.3.1 software (Bradbury et al., 2007) using a Mixed Linear Model (MLM). Population structure was used as a fixed effect in the model’s fitting, while kinship was used as a random effect that was considered by the first two principal components (Patterson et al., 2006; Price et al., 2006).

### Detection of marker trait associations for quality traits

Associations of GA Score, GH, PR Score, Protein %, SDSS Value, and TWt, with candidate loci were identified. We obtained the p-values to determine the significance of the association of traits with the markers and the percent variance explained (PVE), which predicted the extent of the QTL effects. The Manhattan plots for grain quality traits were generated in the GWAS, indicating the most significant associations with a −log10 (P value) greater than 3, along with the Bonferroni correction threshold (we used the Bonferroni correction for multiple testing with an α level of 0.01 for the quality traits and a relaxed α level of 0.20 for all the other datasets) and quantile-quantile (Q-Q) plots.

### Prediction of candidate gene and modeling of homology

The ENSEMBL Wheat database and the International Wheat Genome Sequencing Consortium (IWGSC) RefSeq v1.1 annotations were used to find candidate genes related to the stable loci discovered in this investigation. To find candidate genes, regions within the 1 Mbp window of the localized stable MTA were also chosen. For the gene network analysis and homology finding, an open-source online software, Knetminer, was used at: https://knetminer.org (accessed on Oct 28, 2022) (Hassani-Pak et al., 2021).

## Results

### Phenotypic variation and heritability grain quality traits and agronomic traits

A range of variation for all grain quality traits was reported in the advanced breeding lines of the spring wheat panel. The Protein % ranged from 8.50 to 12.35, with a mean of 10.35 and a CV of 8.61%. Similarly, TWt, GA Score, PR Score, SDSS values, and GH ranged from 66.50 to 78.00, 4.0 to 6.0, 2.20 to 5.50, 29.0 to 50.0, and 7.70 to 12.0 respectively (**Table 1**).

**Table 1:** Descriptive statistics of various quality traits of wheat.

Traits	Range		Mean	SD	CV%
	Minimum	Maximum			
Protein %	8.50	12.35	10.35	0.89	8.61
TWt	66.50	78.00	73.95	2.00	2.70
GA Score	4.00	6.00	5.41	0.30	5.51
PR Score	2.20	5.50	3.25	0.42	12.82
SDSS Value	29.00	50.00	40.03	4.46	11.15
GH	7.70	12.00	9.60	0.97	10.06

Standard deviation (SD), Coefficient of variance (CV%); Grain Appearance Score (GA Score), Grain Hardness (GH), Phenol Reaction Score (PR Score), Protein Percentage (Protein %), SDS Sedimentation Value (SDSS Value), Test Weight (TWt).

Concerning the quantitative traits analysis, a range of variation was observed for GRYLD and other yield related traits at both the locations (Ludhiana and Jabalpur). The broad-sense heritability for the traits under consideration ranged from 0.40 to 0.91 (**Table 2**). The highest broad-sense heritability (0.91) was observed for DTHD at Jabalpur, while the same for GRYLD at Ludhiana and Jabalpur were recorded as 0.69 and 0.60, respectively. Similarly, nearly stable and high heritability were observed for TKW at Ludhiana (0.82) and Jabalpur (0.74). An excellent yielding line (GID: 6692345; SOKOLL/3/PASTOR//HXL7573/2*BAU/4/SOKOLL/WBLL1) with a yield of >7.0 t/h was observed for the Ludhiana location, while there were three lines (GID: 6681676, QUAIU#1/SUP152; GID: 6681793, ND643/2*WBLL1/4/WHEAR/KUKUNA/3/C80.1/3*BATAVIA//2*WBLL1 and GID: 6681817 SUP152/QUAIU#2) were >7.8 t/h for the Jabalpur location. In grain quality traits, the lines with GID 6568703 (PRL/2*PASTOR/4/CHOIX/STAR/3/HE1/3*CNO79//2*SERI*2/5/CHONTE), 6692267 (PASTOR//HXL7573/2*BAU/3/ATTILA/3*BCN/4/SOKOLL/3/PASTOR//HXL7573/2*BAU), and 6692345 (SOKOLL/3/PASTOR//HXL7573/2*BAU/4/SOKOLL/WBLL1) had 12.35, 12.32, and 12.28 protein %, respectively. The lines (GID: 6684333, SWSR22T.B./2*BLOUK #1//WBLL1*2/KURUKU) had high test weight value (78) while three lines (GID: 6568578, KIRITATI/4/2*SERI.1B*2/3/KAUZ*2/BOW//KAUZ/5/2*SUP152, GID: 6568703, PRL/2*PASTOR/4/CHOIX/STAR/3/HE1/3*CNO79//2*SERI*2/5/CHONTE and GID: 6684107, MUTUS*2/HARIL #1) had high grain hardness of 11.8, 11.8, and 12.0, respectively.

**Table 2:** Variability analysis of various yield-related agronomic traits at two locations.

Loc	Traits	H^2^	G Var	R Var	G Mean	LSD	CV
LDH	DTHD	0.83	6.53	2.63	102.11	3.02	1.59
	DAYSMT	0.68	1.49	4.77	146.80	2.70	1.49
	PH	0.40	2.51	5.13	93.28	2.18	2.37
	TKW	0.82	12.18	5.18	39.41	4.13	5.78
	GRYLD	0.69	0.40	0.37	6.25	1.00	9.68
JBP	DTHD	0.91	9.70	2.01	75.44	2.77	1.88
	DAYSMT	0.62	3.75	4.67	118.39	3.39	1.83
	PH	0.47	5.12	11.62	100.18	4.61	3.40
	TKW	0.74	10.52	7.49	46.40	4.68	5.90
	GRYLD	0.60	0.13	0.17	7.13	0.65	5.86

Loc, location; Env, Environment; H2, heritability; G Var, genotypic variance; R Var, residual variance; LSD, least significant difference; CV, critical variance; LDH, Ludhiana; JBP, Jabalpur. DTHD, days to heading; DAYSMT, days to maturity; GRYLD, grain yield; TGW, thousand-grain weight.

### Correlations between agronomic and quality traits of both locations

**Figure 1:**
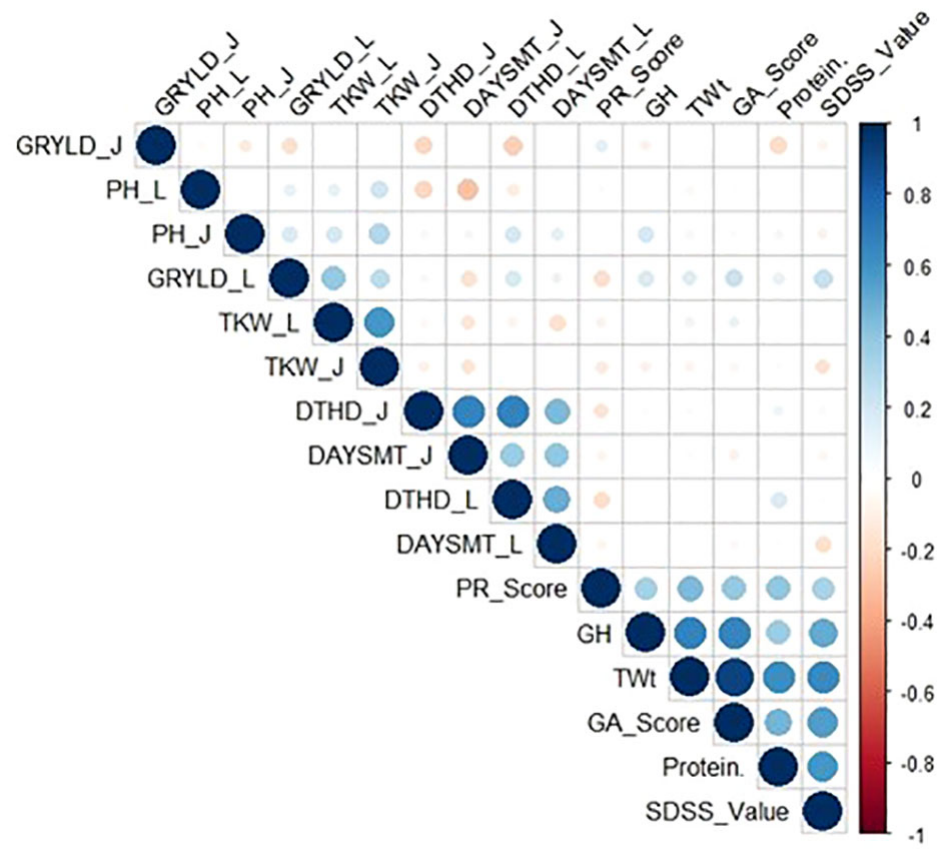
Correlation of agronomic traits in wheat lines. Phenotypic correlations between days to heading (DTHD), days to maturity (DAYSMT), plant height (PH), grain yield (GRYLD), thousand kernel weight (TKW), Ludhiana (_L), Jabalpur (_J), grain appearance score (GA Score), grain hardness (GH), phenol reaction score (PR Score), protein percentage (Protein %), SDS sedimentation value (SDSS Value), and test weight (TWt). The upper and lower 95% confidence intervals are included in parenthesis below the correlation value.

Location-wise correlation among the agronomic traits was analyzed. For Ludhiana and Jabalpur, the suffixes ‘L’ and ‘J’, respectively, have been added to the trait names. GRYLD_L showed a positive correlation with TKW_L, DTHD_L, PH_L, and DAYSMT_L with values of 0.39, 0.17, 0.12, and 0.10, respectively (**Figure 1**; **Supplementary Table 2**). TKW_L showed a positive correlation with PH_L with the value of 0.11 and a negative correlation with DAYSMT_L and DTHD_L with -0.18 and -0.09 values, respectively. GRYLD_J showed a positive (0.04) correlation with DAYSMT_J and TKW_J, while it had a negative correlation with DTHD_J, and PH_J with -0.21 and -0.13, respectively. TKW_J showed a positive correlation with PH_J with a value of 0.30 and a negative correlation with DAYSMT_J and DTHD_J with -0.14 and -0.10 values, respectively.

The correlation between the quality traits revealed that there were positive correlations between all the traits. For example, Protein % has a high correlation with the TWt, GA score, PR score, SDSS value, and GH with the values of 0.62, 0.46, 0.39, 0.59, and 0.36, respectively. Similarly, TWt had a high correlation with the GS score, PR score, SDSS value, and GH with the values of 0.92, 0.45, 0.64, and 0.66, respectively. We observed a positive correlation between SDSS value and HG (0.51). A similar pattern was observed for the GA score with the PR score (0.39), SDSS value (0.56), and GH (0.66) (**Supplementary Table 1**). There was a significant correlation between the GA score and PR score, SDSS value, and GH with values of 0.39, 0.56, and 0.66, respectively. In addition, the PR score correlated well with SDSS and GH with values of 0.33 and 0.34. In addition, SDSS value had a high correlation with GH with a value of 0.51.

The correlation between GRYLD and quality traits across sites (Ludhiana and Jabalpur) elucidated that, GRYLD_L has positive correlation with Protein%, TWt, GA Score, SDSS Value, and GH (0.11, 0.15, 0.23, 0.23 and 0.17, respectively) and showed a negative correlation with the PR Score (-0.18). Furthermore, GRYLD_J had very low correlation with GA Score (0.02) and PR Score (0.12).

### Marker densities and population structure

**Figure 2:**
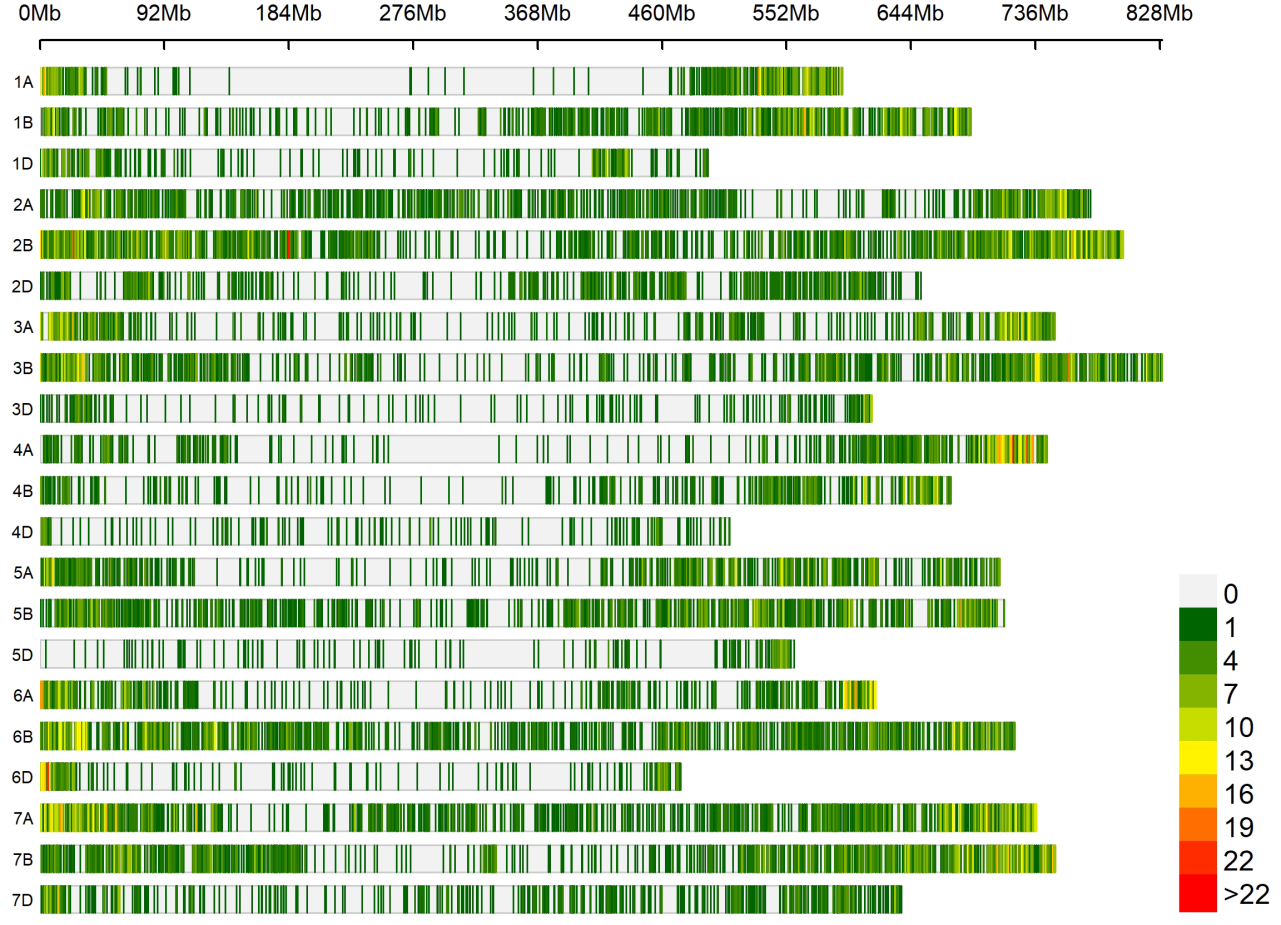
Densities of 18,351 genotyping-by-sequencing markers in the reference bread wheat genome (RefSeq v1.0). The color key with marker densities indicates the number of markers within a window size of 1 Mb.

**Figure 3:**
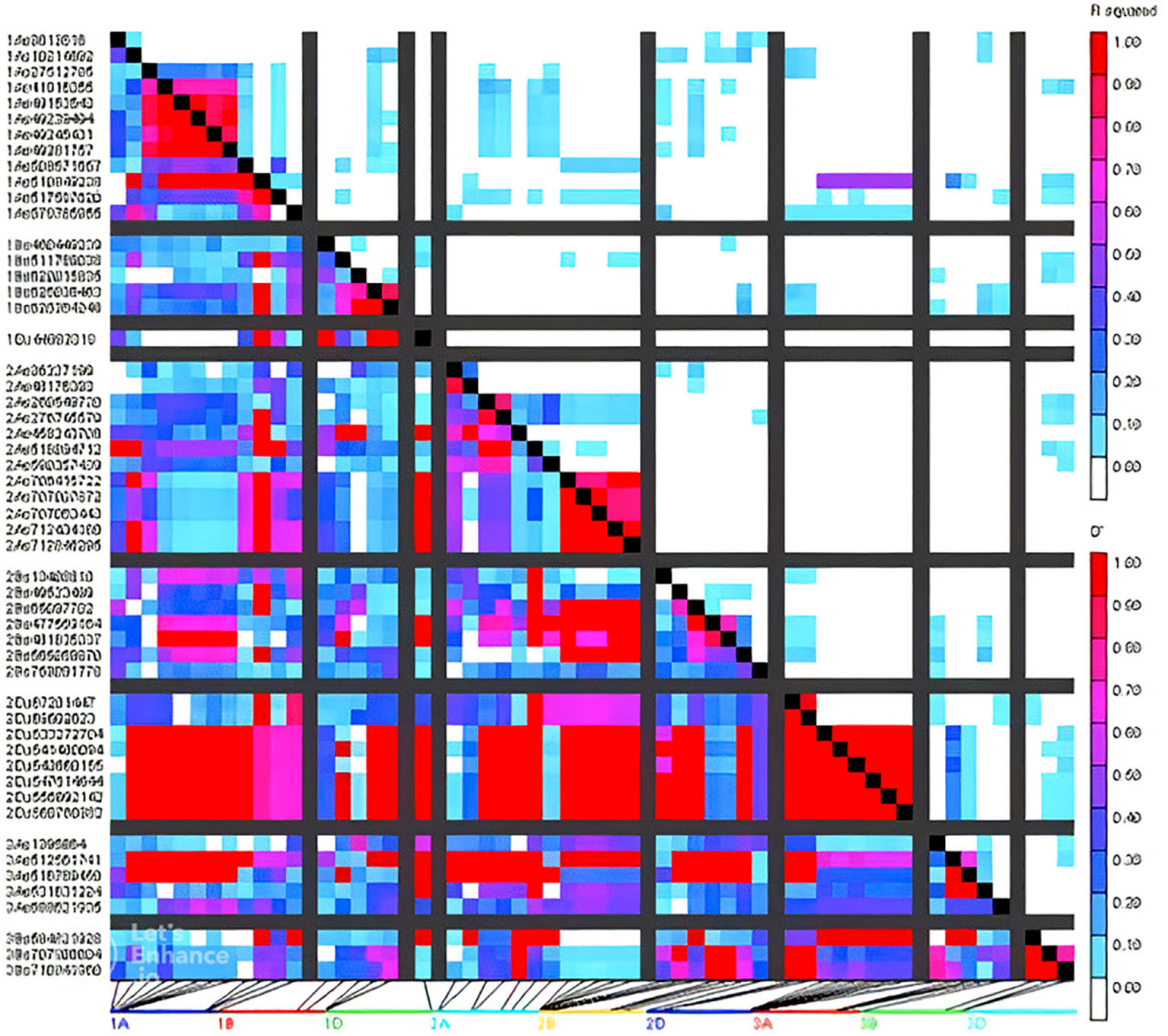
Patterns of LD blocks (right) of GWAS results indicating the position of candidate genes and/or QTL regions associated with grain quality traits and agronomic traits.

**Figure 4:**
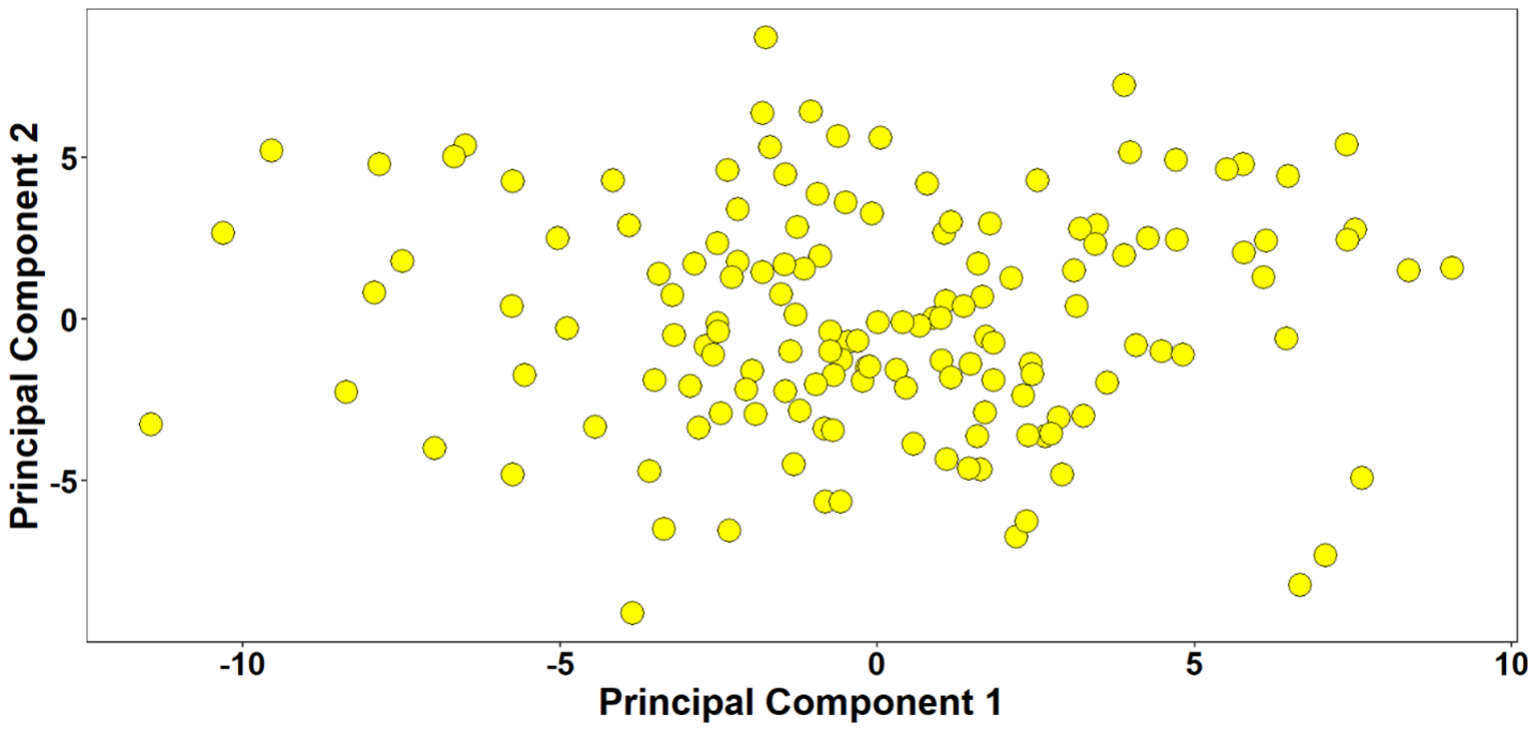
Population structure analysis of 154 lines. The plot of the first two principal components explaining 6.9% and 5.4% of the variation, respectively indicated weak population structure with high relatedness between the lines.

Marker densities of all 18351 GBS markers utilized, aligned to RefSeq v1.0, showed that the telomeric and sub-telomeric regions had higher densities than the centromeric regions in all chromosomes (**Figure 2**). The B-genome has the highest number of markers (48.8%), followed by the A-genome (36.3%) and the D-genome (13.6%). The SNPs in high linkage disequilibrium with one another are reflected by the red area and are consequently inherited together (**Figure 3**). Population structure analysis of all the 154 lines in this study indicated moderate population structure, high diversity, and relatedness between the lines across the locations. The first two principal components plot, PC1 and PC2, explain 6.9% and 5.4% of the variation, respectively (**Figure 4**).

### Detection of marker trait associations for grain quality traits

**Figure 5:**
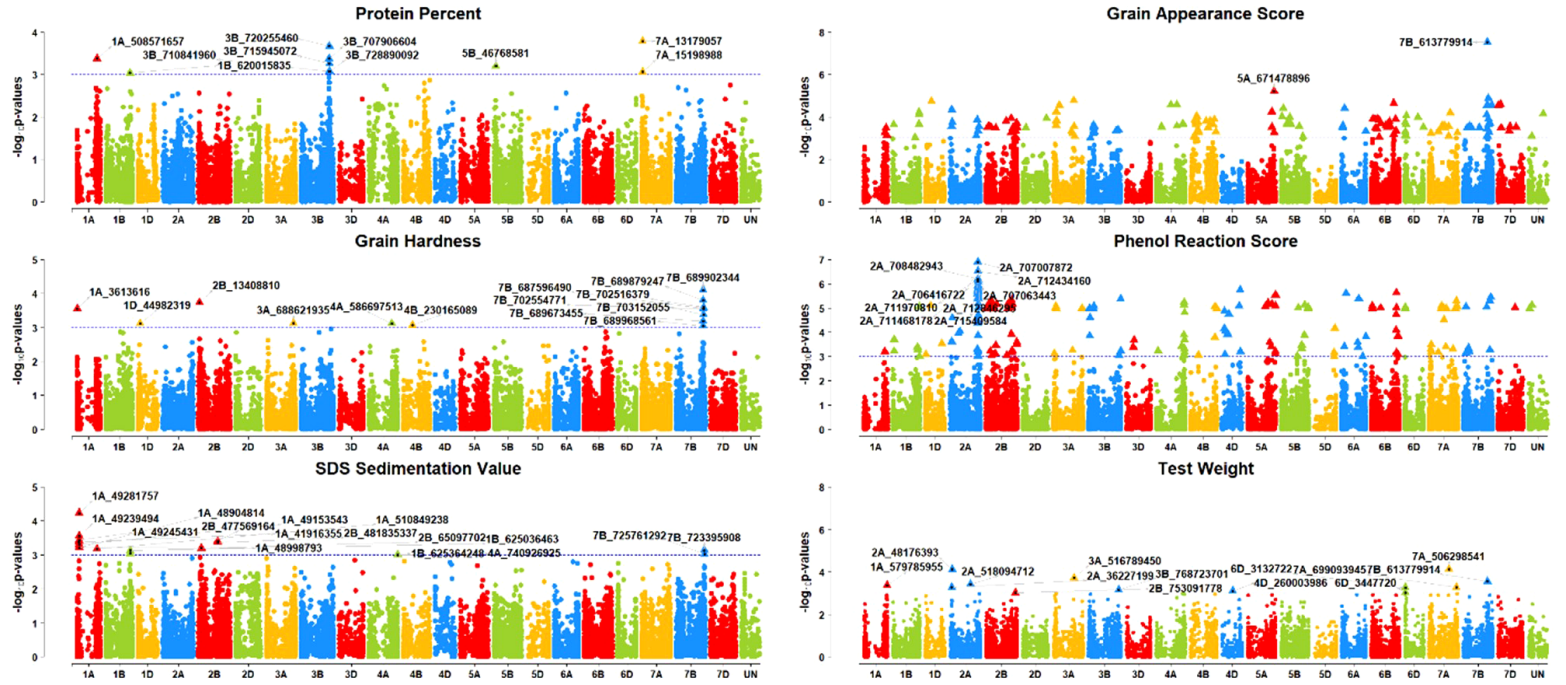
Manhattan plots of GWAS results for grain quality traits (grain appearance score (GA Score), grain hardness (GH), phenol reaction score (PR Score), protein percentage (Protein %), SDS sedimentation value (SDSS Value), and test weight (TWt).); Threshold = −log10(p−value) > 3.

For the quality traits, a total of 55 significantly (*P <*0.001 and Bonferroni correction cut-off value of 0.2) associated SNPs were detected (**Table 3**, **Figure 5**). For GA Score, two SNPs, S5A_671478896 and S7B_613779914, were identified on chromosomes 5A and 7B that explained 18% and 27% of phenotypic variation, respectively. For GH, 13 MTAs were detected on 1A, 1D, 2B, 3A, 4A, 4B, and 7B with 9 – 13% PVE. Interestingly, seven MTAs were on chromosome 7B. Furthermore, SNPs S7B_689902344 and S2B_13408810 had 13% and 12% PVE, respectively. For PR score, five MTAs were detected only on a single chromosome, 2A, with 20–22% PVE, and two SNPs, S2A_707007872 and S2A_707063443, had 22% PVE.

**Table 3:** Genome wide significant associations (R2) of single nucleotide polymorphisms (SNPs) with quality traits in wheat.

Trait	SNP	Chr	Alleles	Position (Mb)	p-Value	add_effect	PVE%
GA Score	S5A_671478896	5A	G/A	671.48	6.17E-06	-1.01E-01	18
	S7B_613779914	7B	G/T	613.78	3.06E-08	3.11E-03	27
GH	S1A_3613616	1A	T/C	3.61	2.85E-04	1.61E-01	11
	S1D_44982319	1D	C/T	44.98	7.71E-04	-7.54E-02	10
	S2B_13408810	2B	A/C	13.41	1.87E-04	-2.28E-01	12
	S3A_688621935	3A	A/G	688.62	7.48E-04	1.18E-01	10
	S4A_586697513	4A	C/A	586.7	7.70E-04	5.62E-01	10
	S4B_230165089	4B	C/T	230.17	8.62E-04	-2.45E-01	9
	S7B_689902344	7B	G/A	689.9	7.99E-05	5.19E-01	13
	S7B_702516379	7B	T/C	702.52	2.55E-04	8.81E-01	11
	S7B_687596490	7B	C/T	687.6	2.69E-04	-5.34E-01	11
	S7B_702554771	7B	A/C	702.55	2.86E-04	-8.81E-01	11
	S7B_703152055	7B	C/A	703.15	4.18E-04	6.25E-01	10
	S7B_689673455	7B	A/G	689.67	6.48E-04	-4.74E-01	10
	S7B_689968561	7B	G/C	689.97	9.12E-04	5.17E-01	9
PR Score	S2A_707007872	2A	T/C	707.01	1.27E-07	-	22
	S2A_707063443	2A	T/C	707.06	1.27E-07	-	22
	S2A_706416722	2A	C/T	706.42	3.06E-07	-	20
	S2A_712434160	2A	T/C	712.43	3.06E-07	-	20
	S2A_712846295	2A	G/C	712.85	3.06E-07	-	20
Protein %	S1A_508571657	1A	A/G	508.57	4.27E-04	-2.81E-01	11
	S1B_620015835	1B	G/A	620.02	9.26E-04	-4.73E-01	10
	S3B_720255460	3B	C/T	720.26	2.17E-04	6.07E-01	12
	S3B_707906604	3B	A/G	707.91	4.22E-04	3.48E-01	11
	S3B_715945072	3B	G/T	715.95	5.56E-04	3.96E-01	11
	S3B_710841960	3B	A/G	710.84	8.53E-04	3.54E-01	10
	S3B_728890092	3B	A/C	728.89	8.60E-04	4.78E-01	10
	S5B_46768581	5B	T/C	46.77	6.35E-04	1.82E-01	11
	S7A_13179057	7A	G/A	13.18	1.65E-04	-4.27E-01	13
	S7A_15198988	7A	C/T	15.2	8.77E-04	-3.57E-02	10
SDSS Value	S1A_49281757	1A	T/C	49.28	5.86E-05	2.87E+00	14
	S1A_49239494	1A	T/A	49.24	2.79E-04	2.80E+00	12
	S1A_49245431	1A	G/A	49.25	3.71E-04	2.70E+00	11
	S1A_41916355	1A	A/G	41.92	4.19E-04	-2.27E+00	11
	S1A_49153543	1A	T/G	49.15	5.04E-04	2.62E+00	11
	S1A_510849238	1A	G/A	510.85	6.65E-04	-2.31E+00	11
	S1B_625036463	1B	A/G	625.04	7.50E-04	-1.73E+00	10
	S1B_625364248	1B	G/T	625.36	9.13E-04	-1.58E+00	10
	S2B_477569164	2B	T/C	477.57	3.96E-04	-1.63E+00	11
	S2B_481835337	2B	G/A	481.84	4.30E-04	-1.96E+00	11
	S2B_65097702	2B	G/A	65.1	6.34E-04	2.35E+00	11
	S4A_740926925	4A	C/A	740.93	9.91E-04	-1.30E+00	10
	S7B_723395908	7B	C/G	723.4	7.31E-04	-8.09E-01	10
TWt	S1A_579785955	1A	A/G	579.79	4.32E-04	8.01E-01	11
	S2A_48176393	2A	C/T	48.18	7.72E-05	3.13E-01	14
	S2A_518094712	2A	C/T	518.09	3.87E-04	1.27E+00	12
	S2A_36227199	2A	G/C	36.23	5.41E-04	-3.66E-01	11
	S2B_753091778	2B	G/C	753.09	9.66E-04	8.16E-01	10
	S3A_516789450	3A	G/C	516.79	1.91E-04	-4.34E-01	13
	S3B_768723701	3B	G/A	768.72	7.02E-04	5.95E-01	11
	S6D_3132722	6D	A/G	3.13	5.44E-04	6.73E-01	11
	S6D_3447720	6D	A/G	3.45	9.43E-04	6.97E-01	10
	S7A_506298541	7A	A/G	506.3	7.51E-05	-1.40E-01	14
	S7A_699093945	7A	A/G	699.09	5.32E-04	-5.44E-01	11
	S7B_613779914	7B	G/T	613.78	2.78E-04	2.38E-01	12

Grain Appearance Score (GA Score), Grain Hardness (GH), Phenol Reaction Score (PR Score), Protein Percentage (Protein %), SDS Sedimentation Value (SDSS Value), Test Weight (TWt), Percent Variance Explained (PVE).

For the Protein %, ten MTAs were obtained on chromosomes 1A, 1B, 3B, 5B, and 7A with 10- 13% PVE, whereas 3A consisted of five MTAs alone. SNP S7A_13179057 had the highest PVE at 13% and S3B_720255460 had 12% PVE. Furthermore, 13 MTAs were found for the SDSS value on chromosomes 1A, 1B, 2B, 4A, and 7B with a range of 10–14% PVE, with a maximum of six MTAs on the 1A chromosome. SNP S1A_49281757 had the highest PVE, 14%, followed by the SNP S1A_49239494 with 12% PVE on the same chromosome. For the TWt, 12 MTAs were reported on chromosomes 1A, 2A, 2B, 3A, 3B, 6D, 7A, and 7B with 10–14% PVE.SNPs S2A_48176393 and S7A_506298541 had the highest (14%) PVE.

### Detection of marker trait associations for agronomic traits

**Figure 6:**
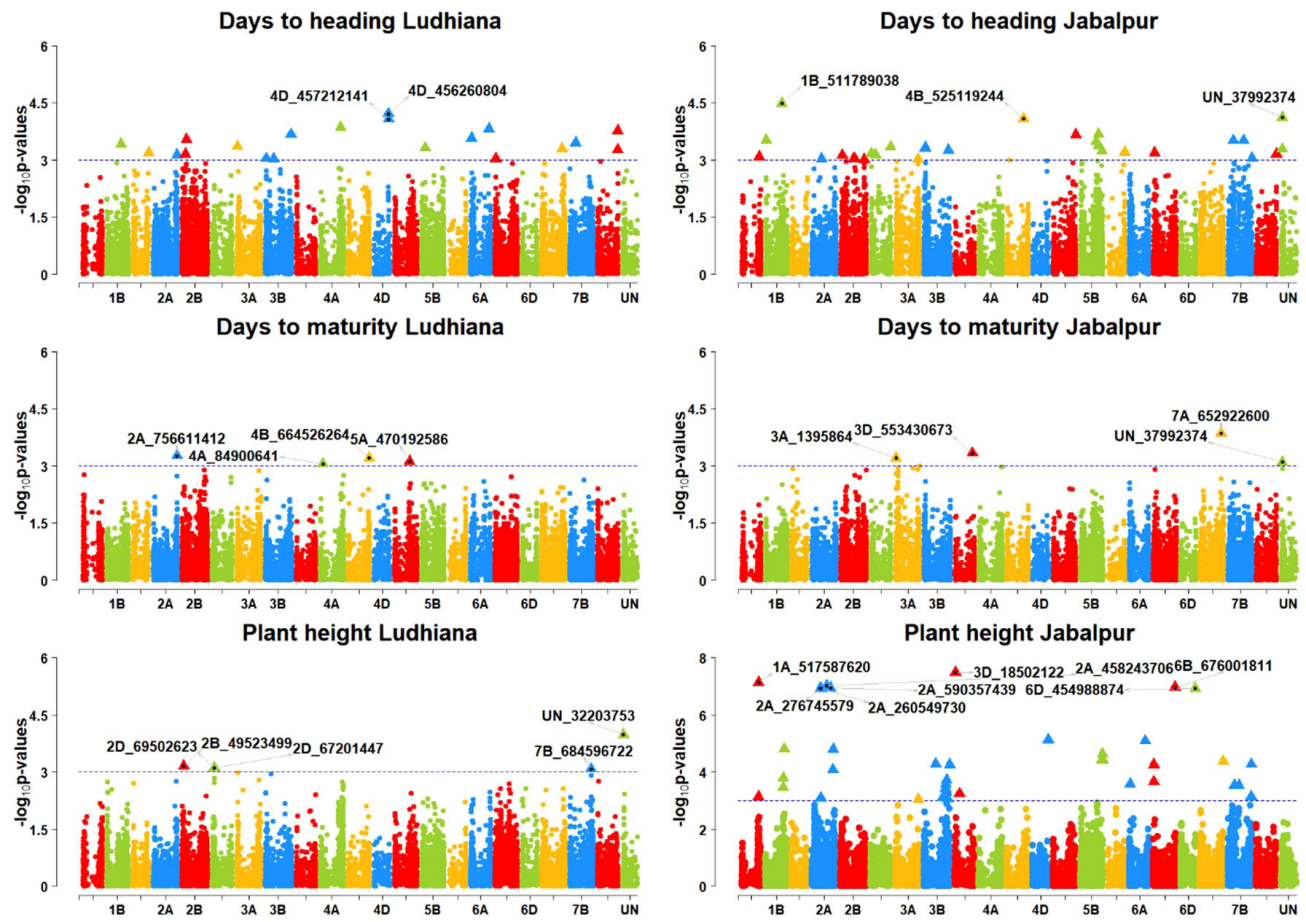
Manhattan plots of GWAS results for agronomic traits days to heading (DTHD), days to maturity (DAYSMT), and grain yield (GRYLD); Ludhiana (LDH), Jabalpur (JBP); Threshold = −log10(p−value) > 3.

**Figure 7:**
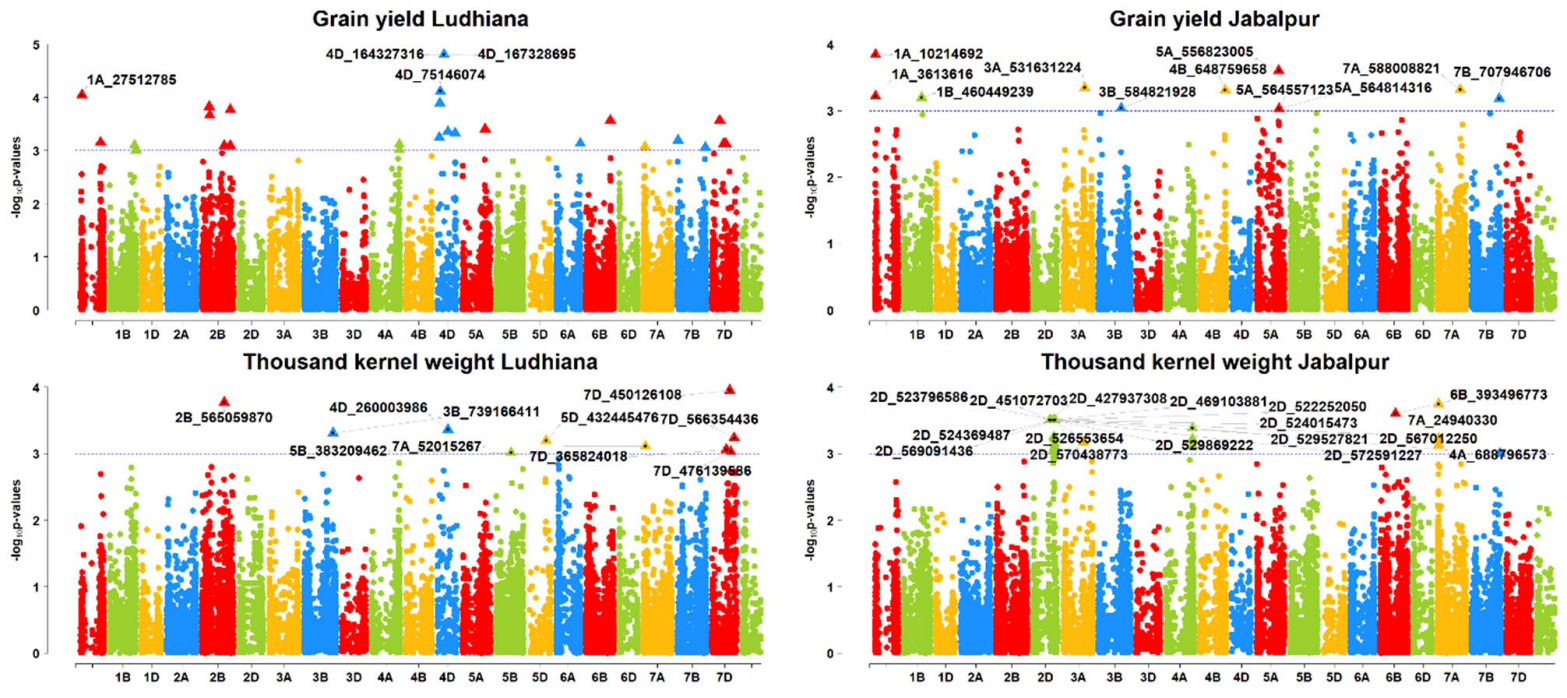
Manhattan plots of GWAS results for agronomic traits grain yield (GRYLD) and thousand kernel weight (TKW); Ludhiana (LDH), Jabalpur (JBP); Threshold = −log10(p−value) > 3.

For the Ludhiana location, a total of 20 MTAs were detected for all the agronomic traits (**Table 4**). Three MTAs, S4A_84900641, S4B_664526264, and S5A_470192586, on chromosomes 4A, 4B, and 5A, respectively, were found for the DAYSMT_L with a range of 10–11% of PVE (**Figures 6**, **7**). Only two MTAs, S4D_456260804a and S4D_457212141, on single chromosome 4D were obtained for the DTHD_L with 14% PVE. Five MTAs were found on chromosomes 2B, 2D, 7B, and UN (non-confirmed location) for PH_L with a range of 10–14% PVE. SNP SUN_32203753 had the highest PVE of 14%, and SNPs S2B_49523499, S2D_69502623, and S2D_67201447 had 11% PVE. Furthermore, eight MTAs were reported for TKW_L with a range of 10–14% PVE on the 2B, 3B, 5B, 7A, and 7D chromosomes where four MTAs shared the 7D chromosome alone. SNPs S7D_450126108 and S2B_565059870 showed a high PVE of 14% and 13%, respectively.

**Table 4:** Genome wide significant associations (R2) of single nucleotide polymorphisms (SNPs) with agronomic traits in wheat at Ludhiana.

Trait	SNP	Chr	Allele	Position (Mb)	p-Value	Add effect	PVE%
DAYSMT_L	S4A_84900641	4A	C/T	84.901	8.87E-04	0.76	10
	S4B_664526264	4B	G/A	664.526	6.20E-04	-0.45	11
	S5A_470192586	5A	T/G	470.193	7.63E-04	0.38	10
DTHD_L	S4D_456260804	4D	C/G	456.261	6.23E-05	1.14	14
	S4D_457212141	4D	C/T	457.212	8.54E-05	1.13	14
GRYLD_L	S1A_27512785	1A	G/A	27.513	9.09E-05	-0.23	13
	S4D_75146074	4D	C/T	75.146	7.72E-05	0.55	14
PH_L	S2B_49523499	2B	C/A	49.523	7.07E-04	-0.17	11
	S2D_69502623	2D	T/C	69.503	7.81E-04	-0.14	11
	S2D_67201447	2D	T/G	67.201	7.85E-04	-0.14	11
	S7B_684596722	7B	T/C	684.597	8.40E-04	-0.27	10
	SUN_32203753	UN	A/T	32.204	1.05E-04	0.04	14
TKW_L	S2B_565059870	2B	T/C	565.06	1.70E-04	0.91	13
	S3B_739166411	3B	C/T	739.166	4.87E-04	-0.33	11
	S5B_383209462	5B	A/G	383.209	9.60E-04	1.11	10
	S7A_52015267	7A	C/T	52.015	7.73E-04	-0.25	11
	S7D_450126108	7D	G/A	450.126	1.12E-04	-1.49	14
	S7D_566354436	7D	T/G	566.354	5.79E-04	-1.93	11
	S7D_365824018	7D	A/G	365.824	8.77E-04	1.68	10
	S7D_476139586	7D	C/T	476.14	9.28E-04	2.41	10

DTHD, days to heading; DAYSMT, days to maturity; GRYLD, grain yield; TKW, thousand-kernel weight; PVE, percent variance explained.

For the Jabalpur location, 44 MTAs were reported for all the agronomic traits (**Table 5**; **Figures 6**, **7**). We found three MTAs for DAYSMT_J on chromosome 7A (S7A_652922600), 3A (S3A_1395864), and 3D (S3D_553430673) which had a maximum of 13% and 11% PVE, respectively, while the location of one MTA that explains 11% of phenotypic variation was not known (SUN_37992374). Similarly, two MTAs for DTHD_J were found on chromosomes 1B (S1B_511789038), with 15% PVE, and 4B (S4B_525119244), with 14% PVE, while the location of one MTA that explained up to 14% of phenotypic variation was not known (SUN_37992374). Plant height had ten MTAs with a maximum of four on the 2A chromosome, whereas the highest PVE, 27%, was obtained on chromosome 3D (S3D_18502122), followed by 26% PVE on both 1A (S1A_517587620) and 2A (S2A_458243706). For TKW_J, a total of 16 MTAs was observed; a maximum of six were on chromosome 2D, followed by four on Chromosome 4A, and three on chromosome 7A. The highest PVE (13%) was obtained for the SNP S7A_24940330, followed by S6B_393496773 with 12% PVE; the rest of the MTAs were in the range of 10–11% for PVE.

**Table 5:** Genome wide significant associations (R^2^) of single nucleotide polymorphisms (SNPs) with agronomic traits in wheat at Jabalpur.

Trait	SNP	Chr	Alleles	Position (Mb)	p-Value	add_effect	PVE%
DAYSMT_J	S3A_1395864	3A	C/T	1.4	6.30E-04	0.67	11
	S3D_553430673	3D	C/A	553.43	4.63E-04	0.64	11
	S7A_652922600	7A	C/G	652.92	1.41E-04	0.61	13
	SUN_37992374	UN	C/T	37.99	7.97E-04	-1.16	10
DTHD_J	S1B_511789038	1B	C/T	511.79	3.30E-05	-0.38	15
	S4B_525119244	4B	G/A	525.12	8.35E-05	0.99	14
	SUN_37992374	UN	C/T	37.99	7.65E-05	-2.48	14
GRYLD_J	S1A_10214692	1A	T/A	10.21	1.42E-04	0.04	13
	S1A_3613616	1A	T/C	3.61	6.04E-04	0.02	11
	S1B_460449239	1B	G/A	460.45	6.39E-04	0.01	11
	S3A_531631224	3A	C/T	531.63	4.48E-04	0.09	11
	S3B_584821928	3B	G/T	584.82	9.19E-04	0.08	10
	S4B_648759658	4B	A/G	648.76	4.95E-04	0.05	11
	S5A_556823005	5A	G/A	556.82	2.50E-04	-0.23	12
	S5A_564814316	5A	A/G	564.81	9.41E-04	0.11	10
	S5A_564557123	5A	T/C	564.56	9.41E-04	-0.15	10
	S7A_588008821	7A	A/G	588.01	4.84E-04	-0.04	11
	S7B_707946706	7B	G/A	707.95	6.58E-04	0.17	11
PH_J	S1A_517587620	1A	G/T	517.59	7.44E-08	0.21	26
	S2A_458243706	2A	G/A	458.24	9.68E-08	-0.19	26
	S2A_590357439	2A	C/T	590.36	1.14E-07	-0.11	25
	S2A_276745579	2A	C/T	276.75	1.20E-07	0.05	25
	S2A_260549730	2A	A/G	260.55	1.21E-07	0.02	25
	S3D_18502122	3D	T/C	18.5	3.34E-08	0.62	27
	S4D_488665508	4D	A/C	488.67	7.76E-06	-0.25	18
	S6A_493375057	6A	C/A	493.38	8.32E-06	-2.46	18
	S6B_676001811	6B	A/G	676	1.10E-07	0.12	25
	S6D_454988874	6D	A/G	454.99	1.19E-07	4.65	25
TKW_J	S2D_533272704	2D	T/C	533.27	5.65E-04	2.86	11
	S2D_560760382	2D	G/A	560.76	6.02E-04	3.1	11
	S2D_556892142	2D	T/C	556.89	7.02E-04	2.99	11
	S2D_541480094	2D	C/A	541.48	8.28E-04	3.01	10
	S2D_547614644	2D	C/T	547.61	8.28E-04	-3.01	10
	S2D_543660155	2D	A/G	543.66	9.11E-04	-2.9	10
	S3A_512561741	3A	A/G	512.56	6.69E-04	-1.71	11
	S4A_688796573	4A	T/C	688.8	4.10E-04	0.54	11
	S4A_688406385	4A	G/C	688.41	6.09E-04	0.65	11
	S4A_679160910	4A	C/G	679.16	6.28E-04	-0.37	11
	S4A_688406519	4A	C/T	688.41	7.54E-04	-0.6	10
	S6B_393496773	6B	T/C	393.5	2.50E-04	-0.36	12
	S7A_24940330	7A	G/A	24.94	1.78E-04	-1.27	13
	S7A_24923657	7A	G/A	24.92	5.95E-04	-1.45	11
	S7A_26061799	7A	A/G	26.06	7.42E-04	1.72	11
	S7B_729805573	7B	G/A	729.81	9.99E-04	-1.27	10

DTHD, days to heading; DAYSMT, days to maturity; GRYLD, grain yield; TKW, thousand-kernel weight, percent variance explained (PVE).

### Detection of marker trait associations for grain yield

For grain yield, only two MTA were detected for the Ludhiana location, SNPs S1A_27512785 (Chromosome 1A) and S4D_75146074 (Chromosome 4D), with 13 and 14% of PVE, respectively (**Figure 7**). At the Jabalpur location, 11 MTAs were obtained for GRYLD with a range of 10–13% PVE. There was a maximum of three MTAs on the chromosome 5A, followed by two on the chromosome 1A; the rest were on single chromosomes such as S1B_460449239 on 1B, S3A_531631224 on 3A, S3B_584821928 on 3B, S4B_648759658 on 4B, S7A_588008821 on 7A, and S7B_707946706 on 7B. Two SNPs, S1A_10214692 on 1A and S5A_556823005, on 5A, had the highest phenotypic variance with 13% and 12% PVE, respectively.

### Candidate gene prediction and associated network

A total of 116 SNPs were physically mapped to IWGSC RefSeqv1.1 with high confidence. To identify the putative candidate genes, the 1Mb flanking region of the mapped SNPs was annotated using EnsemblPlant BioMart. This led to the identification of 19 SNPs overlapped by candidate genes (**Table 6**). Based on the literature survey and current findings, 19 SNPs were considered as novel, and were associated with the following traits: protein %, SDS, PH, DTHD, PR Score, TWt, TKW, GH, DMT, and GYLD. The validation results in KnetMiner network showed that the SNPs for SDSS, such as S1A_49153543, S1A_49281757, S2B_477569164, and S4A_740926925 overlapped with *TraesCS1A02G066900, TraesCS1A02G067300, TraesCS2B02G333900*, and *TraesCS4A02G491100*.

**Table 6:** A list of predicted proteins and function translated by IWGSC genes overlapping 19 Novel SNPs.

SNP_Position	Traits related	IWGSC ID	Predicted protein	Function	References
S1A_49153543	SDSS	TraesCS1A02G066900	SPD1	The SPD1 gene encodes a member of the AAA+ ATPase superfamily involved in plastid development during early seedling growth.	Ruppel et al., 2011
S1A_49281757	SDSS	TraesCS1A02G067300	RRP5	All results support an involvement of the analyzed proteins in ribosome biogenesis but differences in rRNA processing, gametophyte and embryo development suggested an alternative regulation in plants	Missbach et al., 2013
S2B_477569164	SDSS	TraesCS2B02G333900	Phytosiderophore biosynthesis like DMAS1-B, NRAMP2 and NAAT2-D	Fe/Zn transport and accumulation in grain	Gupta et al., 2020
S2B_753091778	Twt	TraesCS2B02G559000	B30.2/SPRY_sf	The B30.2/SPRY domain in these proteins is likely to function through protein-protein interaction	Woo et al., 2006
S2D_69502623	PH	TraesCS2D02G120100	GEX1/Brambleberry	GEX1 from Arabidopsis is required for correct pollen maturation	Alandete-Saez et al., 2011
S3A_512561741	TKW	TraesCS3A02G284100	Galacturonosyltransferase (GAUT)	GAUTs are involved in pectin and xylan biosynthesis	Bouton et al., 2002
S3A_531631224	GYLD	TraesCS3A02G296900	PyrdxlP-dep_Trfase_dom1	PLP-dependent enzymes are primarily involved in the biosynthesis of amino acids and amino acid-derived metabolites, but they are also found in the biosynthetic pathways of amino sugars and in the synthesis or catabolism of neurotransmitters; pyridoxal phosphate can also inhibit DNA polymerases and several steroid receptors	Mozzarelli and Bettati., 2006
S3B_720255460	P%	TraesCS3B02G471800	LRR_dom_sf/NB-ARC	Proteins containing LRRs include tyrosine kinase receptors, cell-adhesion molecules, virulence factors, and extracellular matrix-binding glycoproteins, and are involved in a variety of biological processes, including signal transduction, cell adhesion, DNA repair, recombination, transcription, RNA processing, disease resistance, apoptosis, and the immune response	van der Biezen and Jones., 1998
S3B_728890092	P%	TraesCS3B02G481200	F-box-like_dom_sf/F-box_dom	First identified in cyclin-F as a protein-protein interaction motif, the F-box is a conserved domain that is present in numerous proteins with a bipartite structure	Bai et al., 1996
S3B_739166411	TKW	TraesCS3B02G494600	Conserved oligomeric Golgi complex, subunit 4 (COG_su4)	COG4 is a component of the conserved oligomeric Golgi (COG) complex which mediates the proper glycosylation of proteins trafficking through the Golgi apparatus. It is included in the CATCHR (complexes associated with tethering containing helical rods) family, which includes components of the exocyst, GARP, and DSL1 complexes and share structural and functional features: the α-helical bundles at the middle/C-terminal (described as domains A-D/E) and a N-terminal coiled-coil region.	Santana-Molina et al., 2021
S3B_768723701	Twt	TraesCS3B02G526500	Bax_inhibitor_1-related	BI-1 also regulates cell death triggered by ER stress. BI-1 appears to exert its effect through an interaction with calmodulin	Weis et al., 2013
S4A_679160910	TKW	TraesCS4A02G406300	DHNA_phytyltransferase_MenA	2-carboxy-1,4-naphthoquinone phytyltransferase (IPR011937)	Johnson et al., 2000
S4A_740926925	SDSS	TraesCS4A02G491100	Thioredoxin-like_sf	Several biological processes regulate the activity of target proteins through changes in the redox state of thiol groups (S2 to SH2), where a hydrogen donor is linked to an intermediary disulphide protein. Such processes include the ferredoxin/thioredoxin system, the NADP/thioredoxin system, and the glutathione/glutaredoxin system. Several of these disulphide proteins share a common structure, consisting of a three-layer α/β/α core. Proteins that contain domains with a thioredoxin-like fold	Buchanan and Balmer., 2005
S4D_488665508	PH	TraesCS4D02G330500	Helix-loop-helix DNA-binding domain superfamily (HLH_DNA-bd_sf)	A number of eukaryotic proteins, which probably are sequence specific DNA-binding proteins that act as transcription factors, share a conserved domain of 40 to 50 amino acid residues. The proteins of this subfamily act together with co-repressor proteins, like groucho, through their -terminal motif WRPW.	Murre et al., 1989
S5A_556823005	GYLD	TraesCS5A02G354200	Not available	Not available	Not available
S7A_13179057	P%	TraesCS7A02G031700	Aminoacyl-tRNA synthetase, class II (D/K/N) (IPR004364)	The aminoacyl-tRNA synthetases (also known as aminoacyl-tRNA ligases) catalyze the attachment of an amino acid to its cognate transfer RNA molecule in a highly specific two-step reaction. These proteins differ widely in size and oligomeric state, and have limited sequence homology.	Woese et al., 2000
S7A_652922600	DAYSMT	TraesCS7A02G458100	Znf_RING/FYVE/PHD	Znf-containing proteins function in gene transcription, translation, mRNA trafficking, cytoskeleton organization, epithelial development, cell adhesion, protein folding, chromatin remodeling and zinc sensing, to name but a few	Matthews and Sunde., 2002
S7B_689968561	GH	TraesCS7B02G420600	Peptidase S28/Alpha/Beta hydrolase fold	Serine carboxypeptidase S28 family comprises carboxypeptidase PRCP and the aminopeptidase DPP7. The cap domain (SKS) is formed by 11 α-helices and two strands interconnected by loops. It contains four disulphide bonds which are assumed to be involved in stabilizing the structure. The SKS domain is a rare fold possibly present only in the S28 serine peptidase family.	Bezerra et al., 2012
S7D_476139586	TKW	TraesCS7D02G367800	Major intrinsic protein (IPR000425)	The major intrinsic protein (MIP) family is large and diverse, possessing over 100 members that form transmembrane channels. These channel proteins function in water, small carbohydrate (e.g., glycerol), urea, NH3, CO2 and possibly ion transport, by an energy independent mechanism.	Fu et al., 2000

The SNPs were associated with coding proteins such as SPD1 (involved in plastid development during early seedling growth); RRP5 (role in alternative regulation in plants); DMAS1-B, NRAMP2 and NAAT2-D (Fe/Zn transport and accumulation in grain); and Thioredoxin-like_sf (Redox regulation) (**Supplementary Figure 1**). The SNPs for TKW (S3A_512561741, S3B_739166411, S4A_679160910, and S7D_476139586) overlapped with *TraesCS3A02G284100, TraesCS3B02G494600, TraesCS4A02G406300*, and *TraesCS7D02G367800.* These *Traes IDs* code proteins such as GAUT (involved in pectin and xylan biosynthesis); COG_su4 (mediates the proper glycosylation of proteins trafficking through the Golgi apparatus); DHNA_phytyltransferase_MenA (involved in 2-carboxy-1,4-naphthoquinone phytyltransferase); and MIP (These channel proteins function in water, small carbohydrate (e.g., glycerol), urea, NH3, CO2, and possibly ion transport). Likewise, the SNPs for protein %, viz. S3B_720255460, S3B_728890092, and S7A_13179057, were found to be associated with *TraesCS3B02G471800, TraesCS3B02G481200*, and *TraesCS7A02G031700* respectively, which code for LRR_dom_sf/NB-ARC (involved in a variety of biological processes); F-box-like_dom_sf/F-box_dom (present in numerous proteins with a bipartite structure); and Aminoacyl-tRNA synthetase (These channel proteins function in water, small carbohydrate (e.g., glycerol), urea, NH3, CO2 and possibly ion transport, by an energy independent mechanism). Similarly, for TWt, PH, GYLD, and DAYSMT, we found 2, 2, 2, and 1 overlapped genes, respectively (see detail in **Table 6**).

## Discussion

The performance of a wheat crop should not be judged only from the angle of grain yield as it has several end-product qualities that determine the market value with different value-added parameters. We must explore the different combinations of additional value-added quality parameters to select for desirable end-use quality. Molecular markers linked to the desirable traits is a holistic approach and can be applied in molecular breeding as a tool to identify varieties and lines at any crop development stage. This study attempted the high-resolution genetic dissection of quality, yield, and agronomic variables in spring wheat to find new valuable alleles in genotypes.

The heritability of GRYLD and TKW was good, while for the phenological traits, it was high for DTHD and moderate for DAYSMT and PH. This implies that the phenotypic measurements were of very high quality and that the attributes had a high degree of predictive power. It was reported (Maphosa et al., 2014; Würschum et al., 2018) that GRYLD, a highly quantitative and environmentally sensitive trait, showed significant variation among environments. We also found that agronomic traits significantly contributed to variance explanation and that their heritability was lower than that of other factors, indicating a considerable G×E impact on GRYLD. Therefore, moderate heritability values for GRYLD were anticipated, given that multiple genes govern it. The lower sowing density with smaller plots may also impact the low heritability and yield variances (Thorwarth et al., 2017; Bhatta et al., 2018). The two locations used in this study have very distinct climates. Due to the high ambient temperature, the growing season is comparatively shorter in Jabalpur than in Ludhiana, which has a significantly colder environment with longer growing seasons (Mondal et al., 2016).

Grain quality is a cumulative effect of several traits such as grain protein content, grain hardness, GA score, PR score, SDSS value, and test weight. There is a perception that for good end-products and chapati-making quality, there is a specific combination of the desired grain quality features. To ascertain which combination of quality has what relationship with the others, we estimated correlations between the grain quality of the advanced breeding lines. Likewise, we proceeded with the correlation study to elucidate the correlation between the agronomic and quality traits. There are only a few reports where correlations between the end-use quality traits such as protein %, TWt, GA Score, PR Score, GH, and SDSS value in spring wheat in multi-environment were studied (Guzmán et al., 2016; Ibba et al., 2020; Tsenov et al., 2020).

Grain protein is the primary determinant of wheat quality, its end use, and commercial value (Cox et al., 1985). However, it is well known that grain protein is negatively correlated with grain yield in wheat; in our study, too, we found that there was a negative correlation between protein percentage and the yield from Jabalpur (-0.20), but there was a slight non-significant correlation between protein % and the yield from Ludhiana (0.11). This revealed the influence of environment on the genotype quality. Despite this negative correlation, many reports have reported simultaneous improvements in grain yield and GP (Niu et al., 2010; Vishwakarma et al., 2014, 2016). Protein % has a positive correlation with all the quality measures. Even in this study, we found that all the quality traits studied significantly correlated. Mladenov et al. (2012) also reported a significant positive correlation between the Protein % and sedimentation (SDSS value). In addition to this, test weight is a measure of grain density, which showed a significantly positive correlation with Protein %; in contrast, none of the other traits had a significant correlation. This elucidates that the previous study’s test weight had shown a positive correlation with TKW but not with the Protein % and SDSS value due to these being environment-specific (Mladenov et al., 2012). Grain yield showed a positive correlation (0.17*) with days to maturity indicating that an increase in days to maturity would increase grain yield as also mentioned by Semnaninejad et al. (2021). In addition to this, Grain yield significantly positively correlated with TKW_L (0.39***) and TKW_J (0.04) for both locations as identified by earlier reports (Semnaninejad et al., 2021; Zhang et al., 2021).

### GWAS for agronomic traits

For days to heading, we identified two MTAs on 4D. For the Jabalpur location, it was on chromosome 1B, 4B UN, which was earlier reported by Cadalen et al. (1998) in a double haploid population with his model that explained that the heading date loci from chromosomes 4B and 4D (Xfba1- 4B, Xglk556–4B, and Xfba211–4D) had the main effects. There were interaction effects with plant height QTLs (Xfba393–1A and Xcdo1188–1B) which explained about 50% of the plant height variation. Worland (1996) elucidated that almost all chromosomes carry genes for heading. This notwithstanding, the important genes *Vrn* (vernalization) and *Ppd* (photoperiod), located in homeologous groups 5 and 2, have a significant role in heading date. The SNPs significantly associated with plant height were identified on 2B, 2 (2D), 7B, and UN for the Ludhiana location and 1A, 2A, 3D, 4D, 6A, 6B, and 6D for the Jabalpur location. Previously, plant height was also reported on chromosomes 1A (Sukumaran et al., 2015), 2A (Ain et al., 2015; Mengistu et al., 2016; Sheoran et al., 2019), and 2B (Zanke et al., 2014; Ain et al., 2015; Gao et al., 2015; Sheoran et al., 2019). In addition, the SNPs identified for PH on chromosome 2B (565.060 Mb) were found in proximity to the reduced height genes Rht4 (609.3 Mb). We also obtained 4 SNPs on chromosome 2A, where the Rht7 gene was reported. In this study, SNPs for PH were detected on chromosome 6A. The locus on 6A was consistently detected under drought, heat, and irrigated conditions for yield (Edae et al., 2015; Lopes et al., 2015). Sukumaran et al. (2015) reported the PH in the WAMI population and PH was not correlated with YLD according to the genetic and phenotypic correlation study, demonstrating that the loci on 6A have pleiotropic effects on several characteristics (Sukumaran et al., 2015). However, numerous studies have shown that QTLs influenced by environmental factors in various crops regulate plant height and heading date (Xu et al., 2005; Zhang et al., 2009). The SNPs significantly associated with DTM were identified on chromosomes 4A, 4B, and 5A for the Ludhiana location and 3A, 3D, and 7A for the Jabalpur location, corresponding to the earlier reported genomic regions for DTM on chromosome 5A (Gahlaut et al., 2019; Sheoran et al., 2019), 4B (Sukumaran et al., 2015), and 7A (Adhikari et al., 2020). SNP S7A_652922600 (*TraesCS7A02G458100*) for the DAYSMT on chromosome 7A plays a key role in the function of Znf-containing proteins.

We found a set of three markers for TKW on chromosome 7A in the region from 718 to 735 Mb while Rathan et al. (2022) and Jamil et al. (2019) also identified markers for TKW on the same chromosomal region at 731.8 Mb in multiple environments. This indicated that this chromosome region might have some haplotype block for the TKW.

A complicated quantitative feature, grain yield contains MTAs dispersed across several chromosomes (Jamil et al., 2019). For grain yield in our study, we found MTAs on chromosomes 1A and 4D for the Ludhiana location, while for the Jabalpur location there were three MTAs on chromosome 5A and two MTAs on 1A, 1B, and 3A. Jamil et al. (2019) reported QTLs on 1A, 1B, 5A, and 3A for GRYLD. In an earlier study, two MTAs were present on chromosome 1A with 13% PVE. While chromosome 1B QTLs had 7.55% PVE in our study, one common MTA (S1A_3613616) on 1A had a pleiotropic effect with grain hardness that had a positive correlation with GRYLD also, which intimated that GH had a direct effect on GRYLD. Three markers on 1B explained 13% to 16% PVE for GRYLD (Jamil et al., 2019). Moreover, we reported one MTA on the 4D chromosome. Li et al. (2014) also reported a QTL (QGy4D) on the same chromosome flanked by SSR marker Xbarc334-Xwmc331. It is recognized that several important genes regulating plant height, yield productivity, and yield components are located on chromosomes 4B and 4D (Huang et al., 2006). In our study, we used different environments with very distinct climates. Jabalpur has high ambient temperatures in the daytime and cooler nights during the crop growing season, and the crop’s days to heading, flowering, and maturity periods are shorter in comparison to Ludhiana. In contrast, Ludhiana has a significantly colder environment with longer growing seasons (Mondal et al., 2016). These differences in the environment were also seen in the marker-trait association for the agronomic traits at both locations. We assume that this was the main reason for there being no common MTAs identified for the agronomic traits.

### GWAS for quality and values added parameters

For the quality traits, most of the genetics studies undertaken on wheat have used linkage mapping to study the genetic basis of quality determinants. This entails identifying genes/QTLs linked with the trait of interest by establishing linkage disequilibrium (LD) in populations obtained from bi-parental crosses. However, because of the limited number and location of meiotic events, QTL mapping resolution is frequently limited to 10–30 cM, and it can only study a small fraction of the total number of potential alleles in the population from which the parents originated (Zhu et al., 2008). As an alternative to linkage mapping, association mapping (AM) can help locate alleles in a large number of germplasm samples Yu et al., 2006). Earlier investigations revealed that the GLM model could create false-positive sites due to the lack of a Kinship matrix and a shift in the phenotypic interpretation rate (Yu et al., 2006).

Value-added parameters are complex traits influenced by both the genetic background of the germplasm and the growth conditions (Mohan et al., 2022). Earlier genes/QTLs with major and minor effects on wheat end-use quality traits have been identified and characterized. Nonetheless, whether with bi-parental or association studies, genetically dissected the quantitative trait loci (QTLs)/alleles for the GA Score, PR Score, and SDSS Value, these traits remain uncharacterized. The literature search revealed that this is the first time MTAs for GA Score (2), PR score (5), and the SDSS Value (13) have been reported. This novel locus can be helpful in the identification of new end-use quality products with their corresponding combination in the existing germplasm.

The SDSS value is a thorough indicator for subtly assessing wheat quality and one of the crucial tests to gauge flour’s gluten content. This directly affects the flour’s suitability for processing and baking (Peña et al., 2012) Given that the SDSS value is a quantitative variable influenced by genetic and environmental influences, some QTLs can only be found in particular environments. We reported 13 such MTAs, which are consistent with other findings located on chromosomes 1A (6 SNPs), 1B (2), 2B (3), 4A, and 7B (Goel et al., 2019; Zhang et al., 2020; Alemu et al., 2021). We reported SNP S1A_510849238 on 1A, were near to QTL (540,660,000–544,610,000 bp, RefSeqv1.0) as earlier reported by Yang et al. (2020) through a GWAS. In addition, two more QTLs were reported on chromosome 1A QSsv.cau-1A.1.1 (371,573,909–386,426,688 bp, RefSeqv1.0) and QSsv.cau-1A.1.2 (419,490,584–492,004,197 bp, RefSeqv1.0) by (Tian et al., 2021). We found six MTAs associated with SDSS value on chromosome 1A; this indicates that chromosome 1A is an important region for SDSS value.

According to research, grains that react with phenol to generate color also have the unfavorable trait of browning wheat products like pasta and noodles (Bernier and Howes, 1994). This makes grain screening a valuable method for determining the quality of the end product, thus proving useful in screening the end products. For the phenol reaction score, we have reported five MTAz in between 712.85 -706.42 Mb i.e., the 6.43 Mb region only; this elucidated the possibility of a haplotype block for this trait. The phenol color reaction of the grain gene was on the long arm of chromosome 2A. According to Nair and Tomar (2001) *Triticum turgidum* variety *durum* Desf. has at least two genes that regulate the phenol color response.

The GA score was evaluated based on the grain’s size, shape, color, and luster. We have reported only two MTAs for the GA score on 5A and 7B chromosomes. To date, no previous report has been found for the gene/QTLs for GA score. Previously, Kumar et al. (2019) measured six traits related to grain shape and size, namely, length, width, area, length-to-width ratio, test weight, and thousand kernel weight. Despite a significant correlation with grain yield traits, no significant QTL was found for these traits. These findings could lead to the hypothesis that focusing on grain shape and size, particularly an increase in GA, may improve wheat yield by increasing TGW. Test weight is often referred to as the specific weight of a known volume of grain and serves as a crucial quality indicator. We reported MTAs for test weight on chromosomes 1A, 2A (3), 2B, 3A, 3B, 6D (2), 7A (2), and 7B. There are few studies showing QTL for test weight; however, one of the most recent ones found eight loci on chromosomes 1D, 2A, 2B, 2D, 3B, 3D, 4D, and 7A (Cabral et al., 2018), and while another found loci on 1B and 3B (Alemu et al., 2021).

### The absence of Gpc-B1 allows the exploration of the novel identified loci contributed by the lines

Our study did not identify the major Gpc-B1 gene reported on chromosome 6B by Uauy et al. (2006). This indicates that the genotype x environment interactions played a crucial role. Therefore, exploring a non-adapted genotype provides an opportunity to enhance GPC in the cultivated wheat gene pool. *Gpc-B1* played a significant role in developing several lines for the grain protein; however, it was found at par or negative yield (Uauy et al., 2006; Blanco et al., 2012; Vishwakarma et al., 2014, 2016). These independent loci could be useful to enhance GPC through MAS, without compromising yield. In this study, we identified MTAs on chromosomes 1A, 1B, 3B, 5B, and 7A. In earlier reports with bi-parental mapping populations, *QGPC.ndsu.5B* (found on 5BS) and *QGPC.ndsu.7A.2* (found on 7AL) QTLs were present in non-adapted germplasm, according to a comparison with 49 GPC investigations (Kumar A. et al., 2018). Even though El-Feki et al. (2013) discovered a QTL on 5BS, it was too far away from *QGPC.ndsu.5B*. A few previous investigations in both durum (Peleg et al., 2009; Suprayogi et al., 2009) and hexaploid wheat (Mann et al., 2009; Li et al., 2012) found a stable QTL for GPC on 7AL. The QTL *QGPC.ndsu.7A.2* was found near the telomeric end of chromosomal arm 7AL, whereas the QTLs previously published (Mann et al., 2009; Peleg et al., 2009; Suprayogi et al., 2009; Li et al., 2012) were found in the middle of the chromosome arm 7AL. We reported five MTAs on 3B alone within the 20.98 Mb region. The presence of GPC region *QGpc.caas-3B* flanked by marker wmc3-wmc418in in bi-parental mapping that showed a high LOD value, 11.10, with the highest phenotypic variance of 14.5% has been reported previously by Li et al. (2009). This could be of significant interest as these QTLs were independent of grain yield and may be used as haplotype blocks, contributing to the favorable alleles in the future.

### Novel allele for grain hardness on chromosome 7B

Grain hardness or texture in wheat is directly associated with critical end-use quality attributes such as milling yield and flour extraction. Our research corroborated this by indicating a moderately positive relationship between GH and flour extraction in all settings. Grain hardness in wheat is controlled by the main hardness locus (Ha) on chromosome 5DS, which is positioned at a sub-telomeric location (Sourdille et al., 1996; Morris, 2002). Friabilins are 15-kD lipid-binding endosperm-specific proteins encoded by the Ha locus. The two main proteins in friabilins are Puroindoline a (Pina) and Puroindoline b (Pinb) (Gautier et al., 1994). According to a study of diverse wheat sets (Morris, 2002), hard wheat varieties either lack or possess specific mutations for the pin coding genes. The wild-type pin alleles are found in soft wheat types (Bhave and Morris, 2008). In addition to the significance of the Ha gene, previous research has identified numerous additional QTLs linked to hardness (Heo and Sherman, 2013). In this study, we found seven MTAs on 7B, and no QTL for GH on 7B was reported. These MTAs on 7B could be novel alleles, indicating that both parental genotypes will likely contain the Ha locus hardness alleles.

## Conclusion

This is the first study to report a GWAS for value added quality traits in bread wheat *T. aestivum*. Genetic and functional analysis of the associated genomic regions may enhance wheat quality. Overall, several lines with a combination of appropriate grain quality and agronomic traits were identified, especially for protein content that plays a vital role in tackling nutritional deficiencies or hidden hunger. Quality-enriched *T. aestivum* lines and genomic regions harboring grain quality SNPs can accelerate the breeding program for developing nutritional and value-added end product quality wheat varieties.

## Data Availability

The original contributions presented in the study are included in the article/**Supplementary Material**, further inquiries can be directed to the corresponding author/s.

## References

[B1] AACC (2000). Approved Methods of the American Association.

[B2] Adhikari A. Basnet B. R. Crossa J. Dreisigacker S. Camarillo F. Bhati P. K.. (2020). Genome-wide association mapping and genomic prediction of anther extrusion in CIMMYT hybrid wheat breeding program via modeling pedigree, genomic relationship, and interaction with the environment. Front. Genet. 11, 586687. doi: 10.3389/fgene.2020.586687 33363570 PMC7755068

[B3] Ain Q. U. Rasheed A. Anwar A. Mahmood T. Imtiaz M. Mahmood T.. (2015). Genome-wide association for grain yield under rainfed conditions in historical wheat cultivars from Pakistan. Front. Plant Sci. 6, 743. doi: 10.3389/fpls.2015.00743 26442056 PMC4585131

[B4] Alandete-Saez M. Ron M. Leiboff S. McCormick S. (2011). Arabidopsis thaliana GEX1 has dual functions in gametophyte development and early embryogenesis. Plant J. Cell Mol. Biol. 68 (4), 620–632. doi: 10.1111/j.1365-313X.2011.04713.x 21831199

[B5] Alemu A. El Baouchi A. El Hanafi S. Kehel Z. Eddakhir K. Tadesse W.. (2021). Genetic analysis of grain protein content and dough quality traits in elite spring bread wheat (*Triticum aestivum*) lines through association study. J. Cereal Sci. 100, 103214. doi: 10.1016/j.jcs.2021.103214

[B6] Alvarado G. Rodríguez F. M. Pacheco A. Burgueño J. Crossa J. Vargas M.. (2020). META-R: A software to analyze data from multi-environment plant breeding trials. Crop J. 8, 745–756. doi: 10.1016/j.cj.2020.03.010

[B7] Bai C. Sen P. Hofmann K. Ma L. Goebl M. Harper J. W.. (1996). SKP1 connects cell cycle regulators to the ubiquitin proteolysis machinery through a novel motif, the f-box. Cell 86 (2), 263–274. doi: 10.1016/s0092-8674(00)80098-7 8706131

[B8] Bernier A. M. Howes N. K. (1994). Quantification of variation in tyrosinase activity among durum and common wheat cultivars. J. Cereal Sci. 19, 157–159. doi: 10.1006/jcrs.1994.1021

[B9] Bezerra G. A. Dobrovetsky E. Dong A. Seitova A. Crombett L. Shewchuk L. M.. (2012). Structures of human DPP7 reveal the molecular basis of specific inhibition and the architectural diversity of proline-specific peptidases. PloS One 7 (8), e43019. doi: 10.1371/journal.pone.0043019 22952628 PMC3430648

[B10] Bhatta M. Morgounov A. Belamkar V. Baenziger P. S. (2018). Genome-wide association study reveals novel genomic regions for grain yield and yield-related traits in drought-stressed synthetic hexaploid wheat. Int. J. Mol. Sci. 19, 3011. doi: 10.3390/ijms19103011 30279375 PMC6212811

[B11] Bhave M. Morris C. F. (2008). Molecular genetics of puroindolines and related genes: Allelic diversity in wheat and other grasses. Plant Mol. Biol. 66, 205–219. doi: 10.3390/ijms19103011 18049798

[B12] Blanco A. Mangini G. Giancaspro A. Giove S. Colasuonno P. Simeone R.. (2012). Relationships between grain protein content and grain yield components through quantitative trait locus analyses in a recombinant inbred line population derived from two elite durum wheat cultivars. Mol. Breeding 30, 79–92. doi: 10.1007/s11032-011-9600-z

[B13] Bradbury P. J. Zhang Z. Kroon D. E. Casstevens T. M. Ramdoss Y. Buckler E. S. (2007). TASSEL: Software for association mapping of complex traits in diverse samples. Bioinformatics 23, 2633–2635. doi: 10.1093/bioinformatics/btm308 17586829

[B14] Bouton S. Leboeuf E. Mouille G. Leydecker M. T. Talbotec J. Granier F.. (2002). QUASIMODO1 encodes a putative membrane-bound glycosyltransferase required for normal pectin synthesis and cell adhesion in arabidopsis. Plant Cell 14 (10), 2577–2590. doi: 10.1105/tpc.004259 12368506 PMC151237

[B15] Buchanan B. B. Balmer Y. (2005). Redox regulation: a broadening horizon. Annu Rev Plant Biol. 56, 187–220. doi: 10.1146/annurev.arplant.56.032604.144246 15862094

[B16] Cabral A. L. Jordan M. C. Larson G. Somers D. J. Humphreys G. McCartney C. A. (2018). Relationship between QTL for grain shape, grain weight, test weight, milling yield, and plant height in the spring wheat cross RL4452/ ‘AC Domain.’. PloS One 13, e0190681. doi: 10.1371/journal.pone.0190681 29357369 PMC5777647

[B17] Cadalen T. Sourdille P. Charmet G. Tixier M. H. Gay G. Boeuf C.. (1998). Molecular markers linked to genes affecting plant height in wheat using a doubled-haploid population. Theor. Appl. Genet. 96, 933–940. doi: 10.1007/s001220050823

[B18] Cappelli A. Cini E. (2021). Challenges and opportunities in wheat flour, pasta, bread, and bakery product production chains: A systematic review of innovations and improvement strategies to increase sustainability, productivity, and product quality. Sustainability 13, 2608. doi: 10.3390/su13052608

[B19] Carter B. P. Morris C. F. Anderson J. A. (1999). Optimizing the SDS sedimentation test for end-use quality selection in a soft white and club wheat breeding program. Cereal Chem. 76, 907–911. doi: 10.1094/CCHEM.1999.76.6.907

[B20] Cox M. C. Qualset C. O. Rains D. W. (1985). Genetic variation for nitrogen assimilation and translocation in wheat. I. Dry matter and nitrogen accumulation. Crop Sci. 25, 430–435. doi: 10.2135/cropsci1985.0011183X002500030002x

[B21] Doyle J. J. Doyle J. L. (1987). A rapid DNA isolation procedure for small quantities of fresh leaf tissue. Phytochem. Bull. 19, 11–15.

[B22] Edae E. A. Bowden R. L. Poland J. (2015). Application of population sequencing (POPSEQ) for ordering and imputing genotyping-by-sequencing markers in hexaploid wheat. G3 Genes Genomes Genet. 5, 2547–2553. doi: 10.1534/g3.115.020362 PMC4683627 26530417

[B23] El-Feki W. M. Byrne P. F. Reid S. D. Lapitan N. L. V. Haley S. D. (2013). Quantitative trait locus mapping for end-use quality traits in hard winter wheat under contrasting soil moisture levels. Crop Sci. 53, 1953–1967. doi: 10.2135/cropsci2012.12.0674

[B24] Fu D. Libson A. Miercke L. J. Weitzman C. Nollert P. Krucinski J.. (2000). Structure of a glycerol-conducting channel and the basis for its selectivity. Sci. (New York N.Y.) 290 (5491), 481–486. doi: 10.1126/science.290.5491.481 11039922

[B25] Gahlaut V. Jaiswal V. Tyagi B. S. Singh G. Sareen S. Balyan H. S.. (2019). Multi-locus genome wide association mapping for yield and its contributing traits in hexaploid wheat under different water regimes. Sci. Rep. 9, 19486. doi: 10.1038/s41598-019-55520-0 31862891 PMC6925107

[B26] Gao F. Wen W. Liu J. Rasheed A. Yin G. Xia X.. (2015). Genome-wide linkage mapping of QTL for yield components, plant height and yield-related physiological traits in the Chinese wheat cross Zhou 8425B/Chinese spring. Front. Plant Sci. 6, 1099. doi: 10.3389/fpls.2015.01099 26734019 PMC4683206

[B27] Gautier M. F. Aleman M. E. Guirao A. Marion D. Joudrier P. (1994). *Triticum aestivum* puroindolines, two basic cystine-rich seed proteins: cDNA sequence analysis and developmental gene expression. Plant Mol. Biol. 25, 43–57. doi: 10.1007/BF00024197 7516201

[B28] Glaubitz J. C. Casstevens T. M. Lu F.. (2014). TASSEL-GBS: A high capacity genotyping by sequencing analysis pipeline. PloS One 9, e90346. doi: 10.1371/journal.pone.0090346 24587335 PMC3938676

[B29] Goel S. Singh K. Singh B. Grewal S. Dwivedi N. Alqarawi A. A.. (2019). Analysis of genetic control and QTL mapping of essential wheat grain quality traits in a recombinant inbred population. PloS One 14, e0200669. doi: 10.1371/journal.pone.0200669 30840619 PMC6402682

[B30] Güçbilmez Ç. M. Şahin M. Akçacık A. G. Aydoğan S. Demir B. Hamzaoğlu S.. (2019). Evaluation of GlutoPeak test for prediction of bread wheat flour quality, rheological properties and baking performance. J. Cereal Sci. 90, 102827. doi: 10.1016/j.jcs.2019.102827

[B31] Gupta O. P. Pandey V. Saini R. Narwal S. Malik V.K. Khandale T.. (2020). Identifying transcripts associated with efficient transport and accumulation of Fe and Zn in hexaploid wheat (*T. aestivum* L.). J. Biotechnol. 316, 46–55. doi: 10.1016/j.jbiotec.2020.03.015 32305628

[B32] Guzmán C. Ammar K. Govindan V. Singh R. (2019). Genetic improvement of wheat grain quality at CIMMYT. Front. Agric. Sci. Eng. 6, 265–272. doi: 10.15302/J-FASE-2019260

[B33] Guzmán C. Peña R. J. Singh R. Atrique E. Dreisigacker S. Crossa J.. (2016). Wheat quality improvement at CIMMYT and the use of genomic selection on it. Appl. Transl. Genom. 11, 3–8. doi: 10.1016/j.atg.2016.10.004 28018844 PMC5167370

[B34] Hassani-Pak K. Singh A. Brandizi M. Hearnshaw J. Parsons J.D. Amberkar S.. (2021). KnetMiner: a comprehensive approach for supporting evidence-based gene discovery and complex trait analysis across species. Plant Biotechnol. J. 19, 1670–1678. doi: 10.1111/pbi.13583 33750020 PMC8384599

[B35] Heo H. Sherman J. (2013). Identification of QTL for grain protein content and grain hardness from winter wheat for genetic improvement of spring wheat. Plant Breed Biotechnol. 1, 347–353. doi: 10.9787/PBB.2013.1.4.347

[B36] Huang X. Q. Cloutier S. Lycar L.. (2006). Molecular detection of QTLs for agronomic and quality traits in a doubled haploid population derived from two Canadian wheats (*Triticum aestivum* L.). Theor. Appl. Genet. 113, 753–766. doi: 10.1007/s00122-006-0346-7 16838135

[B37] Ibba M. I. Crossa J. Montesinos-López O. A.. (2020). Genome-based prediction of multiple wheat quality traits in multiple years. Plant Genome 13, e20034. doi: 10.1002/tpg2.20034 33217204 PMC12806979

[B38] IWGSC (2018). Shifting the limits in wheat research and breeding using a fully annotated reference genome. Science 80-). 361, 1–163. 10.1126/science.aar7191 30115783

[B39] Jamil M. Ali A. Gul A. Ghafoor A. Napar A. A. Ibrahim A. M. H.. (2019). Genome-wide association studies of seven agronomic traits under two sowing conditions in bread wheat. BMC Plant Biol. 19, 149. doi: 10.1186/s12870-019-1754-6 31003597 PMC6475106

[B40] Jiang Y. Schmidt R. H. Zhao Y. Reif J. C. (2017). Quantitative genetic framework highlights the role of epistatic effects for grain-yield heterosis in bread wheat. Nat. Genet. 49, 1741–1746. doi: 10.1038/ng.3974 29038596

[B41] Johnson T. W. Shen G. Zybailov B. Kolling D. Reategui R. Beauparlant S.. (2000). Recruitment of a foreign quinone into the A(1) site of photosystem i. i. genetic and physiological characterization of phylloquinone biosynthetic pathway mutants in synechocystis sp. pcc 6803. J. Biol. Chem. 275 (12), 8523–8530. 10722690 10.1074/jbc.275.12.8523

[B42] Juliana P. Poland J. Huerta-Espino J. Shrestha S. Crossa J. Crespo-Herrera L.. (2019). Improving grain yield, stress resilience and quality of bread wheat using large-scale genomics. Nat. Genet. 51, 1530–1539. doi: 10.1038/s41588-019-0496-6 31548720

[B43] Kumar A. Jain S. Elias E. M. Ibrahim M. Sharma L. K. (2018). “An overview of QTL identification and marker-assisted selection for grain protein content in wheat,” in Eco-friendly Agro-biological Techniques for Enhancing Crop Productivity. Eds. Sengar R. Singh A. (Springer, Singapore). doi: 10.1007/978-981-10-6934-5_11

[B44] Kumar A. Mantovani E. E. Simsek S. Jain S. Elias E. M. Mergoum M.. (2019). Genome wide genetic dissection of wheat quality and yield related traits and their relationship with grain shape and size traits in an elite × non-adapted bread wheat cross. PloS One 14, e0221826. doi: 10.1371/journal.pone.0221826 31532783 PMC6750600

[B45] Kumar S. Sohu V. S. Gupta S. K. Singh R. P. Bains N. S. (2018). Understanding the chapati making attributes of the Indian wheats – I: The physico-chemical basis. J. Appl. Natural Sci. 10, 572–592. doi: 10.31018/jans.v10i2.1739

[B46] Langmead B. Salzberg S. L. (2012). Fast gapped-read alignment with Bowtie 2. Nat. Methods 9, 357–359. doi: 10.1038/nmeth.1923 22388286 PMC3322381

[B47] Li J. Cui F. Ding Am. Zhao C-h. Wang X-q. Wang L.. (2012). QTL detection of seven quality traits in wheat using two related recombinant inbred line populations. Euphytica 183, 207–226. doi: 10.1007/s10681-011-0448-4

[B48] Li Z. K. Jiang X. L. Peng T. Shi C. L. Han S. X. Tian B.. (2014). Mapping quantitative trait loci with additive effects and additive x additive epistatic interactions for biomass yield, grain yield, and straw yield using a doubled haploid population of wheat (*Triticum aestivum* L.). Genet. Mol. Res. 13, 1412–1424. doi: 10.4238/2014.February.28.14 24634240

[B49] Li Y. Song Y. Zhou R. Branlard G. Jia J. (2009). Detection of QTLs for bread-making quality in wheat using a recombinant inbred line population. Plant Breeding 128, 235–243. doi: 10.1111/j.1439-0523.2008.01578.x

[B50] Liu Y. Lin Y. Gao S. Li Z. Ma J. Deng M. (2017). A genome-wide association study of 23 agronomic traits in Chinese wheat landraces. Plant J. 91, 861–873. doi: 10.1111/tpj.13614 28628238

[B51] Lopes M. S. Dreisigacker S. Peña R. J. Sukumaran S. Reynolds M. P. (2015). Genetic characterization of the wheat association mapping initiative (WAMI) panel for dissection of complex traits in spring wheat. Theor. Appl. Genet. 128, 453–464. doi: 10.1007/s00122-014-2444-2 25540818

[B52] Ma F. Baik B. K. (2018). Soft wheat quality characteristics required for making baking powder biscuits. J. Cereal Sci. 79, 127–133. doi: 10.1016/j.jcs.2017.10.016

[B53] Mann G. Diffey S. Cullis B. Azanza F. Martin D. Kelly A.. (2009). Genetic control of wheat quality: Interactions between chromosomal regions determining protein content and composition, dough rheology, and sponge and dough baking properties. Theor. Appl. Genet. 118, 1519–1537. doi: 10.1007/s00122-009-1000-y 19283360

[B54] Maphosa L. Langridge P. Taylor H. Parent B. Emebiri L. C. Kuchel H.. (2014). Genetic control of grain yield and grain physical characteristics in a bread wheat population grown under a range of environmental conditions. Theor. Appl. Genet. 127, 1607–1624. doi: 10.1007/s00122-014-2322-y 24865506

[B55] Matthews J. M. Sunde M. (2002). Zinc fingers–folds for many occasions. IUBMB Life 54 (6), 351–355. doi: 10.1080/15216540216035 12665246

[B56] Mengistu D. K. Kidane Y. G. Catellani M. Frascaroli E. Fadda C. Pè M. E.. (2016). High-density molecular characterization and association mapping in Ethiopian durum wheat landraces reveals high diversity and potential for wheat breeding. Plant Biotechnol. J. 14, 1800–1812. doi: 10.1111/pbi.12538 26853077 PMC5067613

[B57] Missbach S. Weis B. L. Martin R. Simm S. Bohnsack M. T. Schleiff E. (2013). 40S ribosome biogenesis co-factors are essential for gametophyte and embryo development. PloS One 8 (1), e54084. doi: 10.1371/journal.pone.0054084 23382868 PMC3559688

[B58] Mladenov V. Banjac B. Krishna A. Miloaević M. (2012). Relation of grain protein content and some agronomic traits in European cultivars of winter wheat. Cereal Res. Commun. 40, 532–541. doi: 10.1556/CRC.40.2012.0004

[B59] Mohan D. Sendhil R. Gupta O. P. Pandey V. Krishnappa G. Singh G. P. (2022). Wheat quality index: new holistic approach to identify quality superior genotypes. Cereal Res. Commun. 50, 1105–1115. doi: 10.1007/s42976-022-00254-5

[B60] Mondal S. Rutkoski J. E. Velu G. Singh P. K. Crespo-Herrera L. A. Guzmán C.. (2016). Harnessing diversity in wheat to enhance grain yield, climate resilience, disease and insect pest resistance and nutrition through conventional and modern breeding approaches. Front. Plant Sci. 7. doi: 10.3389/fpls.2016.00991 PMC4933717 27458472

[B61] Morris C. F. (2002). Puroindolines: The molecular genetic basis of wheat grain hardness. Plant Mol. Biol. 48, 633–647. doi: 10.1023/A:1014837431178 11999840

[B62] Mozzarelli A. Bettati S. (2006). Exploring the pyridoxal 5'-phosphate-dependent enzymes. Chem. Rec. (New York N.Y.) 6 (5), 275–287. doi: 10.1002/tcr.20094 17109392

[B63] Murre C. McCaw P. S. Baltimore D. (1989). A new DNA binding and dimerization motif in immunoglobulin enhancer binding, daughterless, MyoD, and myc proteins. Cell 56 (5), 777–783. doi: 10.1016/0092-8674(89)90682-x 2493990

[B64] Nair S. K. Tomar S. M. S. (2001). Genetics of phenol colour reaction of grains and glumes in tetraploid and hexaploid wheats. J. Genet. Breed. 55, 369–373.

[B65] Nakamura K. Taniguchi Y. Taira M. Ito H. (2012). Investigation of soft wheat flour quality factors associated with sponge cake sensory tenderness. Cereal Chem. 89, 79–83. doi: 10.1094/CCHEM-07-11-0081

[B66] Niu N. Arief V. N. Delacy I. H. Lush D. Sheppard J. Zhang G.. (2010). Genetic gain in yield and protein over two cycles of a wheat recurrent selection program. Breed Sci. 60, 181–186. doi: 10.1270/jsbbs.60.181

[B67] Patterson N. Price A. L Reich D. (2006). Population structure and eigenanalysis. PloS Genet. 2, e190. doi: 10.1371/journal.pgen.0020190 17194218 PMC1713260

[B68] Peleg Z. Cakmak I. Ozturk L. Yazici A. Jun Y. Budak H.. (2009). Quantitative trait loci conferring grain mineral nutrient concentrations in durum wheat × wild emmer wheat RIL population. Theor. Appl. Genet. 119, 353–369. doi: 10.1007/s00122-009-1044-z 19407982

[B69] Peña R. J. Trethowan R. Pfeiffer W. H. van Ginkel M. (2012). Quality (end-use) improvement in wheat compositional, genetic, and environmental factors. J. Crop Prod. 5, 1–37. doi: 10.1300/j144v05n01_02

[B70] Poland J. A. Brown P. J. Sorrells M. E. Jannink J. L. (2012). Development of high-density genetic maps for barley and wheat using a novel two-enzyme genotyping-by-sequencing approach. PloS One 7, e32253. doi: 10.1371/journal.pone.0032253 22389690 PMC3289635

[B71] Price A. L. Patterson N. J. Plenge R. M. Weinblatt M. E. Shadick N. A. Reich D. (2006). Principal components analysis corrects for stratification in genome-wide association studies. Nat. Genet. 38, 904–909. doi: 10.1038/ng1847 16862161

[B72] Rathan N. D. Krishna H. Ellur R. K. Sehgal D. Govindan V. Ahlawat A. K.. (2022). Genome-wide association study identifies loci and candidate genes for grain micronutrients and quality traits in wheat (*Triticum aestivum* L.). Sci. Rep. 12, 7037. doi: 10.1038/s41598-022-10618-w 35487909 PMC9054743

[B73] Ray D. K. Mueller N. D. West P. C. Foley J. A. (2013). Yield trends are insufficient to double global crop production by 2050. PloS One 8, e66428. doi: 10.1371/journal.pone.0066428 23840465 PMC3686737

[B74] Ray D. K. Ramankutty N. Mueller N. D. West P. C. Foley J. A. (2012). Recent patterns of crop yield growth and stagnation. Nat. Commun. 3, 1293. doi: 10.1038/ncomms2296 23250423

[B75] Riaz A. Athiyannan N. Periyannan S. K. Afanasenko O. Mitrofanova O. P. Platz G. J.. (2018). Unlocking new alleles for leaf rust resistance in the Vavilov wheat collection. Theor. Appl. Genet. 131, 127–144. doi: 10.1007/s00122-017-2990-5 28980023

[B76] Ruppel N. J. Logsdon C. A. Whippo C. W. Inoue K. Hangarter R. P. (2011). A mutation in arabidopsis seedling plastid development1 affects plastid differentiation in embryo-derived tissues during seedling growth. Plant Physiol. 155 (1), 342–353. doi: 10.1104/pp.110.161414 21045120 PMC3075797

[B77] Santana-Molina C. Gutierrez F. Devos D. P. (2021). Homology and modular evolution of CATCHR at the origin of the eukaryotic endomembrane system. Genome Biol. Evol. 13 (7), evab125. doi: 10.1093/gbe/evab125 34061181 PMC8290106

[B78] Semnaninejad H. Nourmohammadi G. Rameeh V. Cherati A. (2021). Correlation and path coefficient analyses of phenological traits, yield components and quality traits in wheat. Rev. Bras. Engenharia Agricola e Ambiental 25, 597–603. doi: 10.1590/1807-1929/agriambi.v25n9p597-603

[B79] Sheoran S. Jaiswal S. Kumar D. Raghav N. Sharma R. Pawar S.. (2019). Uncovering genomic regions associated with 36 agro-morphological traits in Indian spring wheat using GWAS. Front. Plant Sci. 10, 527. doi: 10.3389/fpls.2019.00527 31134105 PMC6511880

[B80] Shuey W. C. (1960). A wheat sizing technique for predicting flour milling yield. Cereal Sci. Today 5, 71.

[B81] Sourdille P. Perretant M. R. Charmet G. Leroy P. Gautier M. F. Joudrier P.. (1996). Linkage between RFLP markers and genes affecting kernel hardness in wheat. Theor. Appl. Genet. 93, 580–586. doi: 10.1007/BF00417951 24162351

[B82] Sukumaran S. Dreisigacker S. Lopes M. Chavez P. Reynolds M. P. (2015). Genome-wide association study for grain yield and related traits in an elite spring wheat population grown in temperate irrigated environments. Theor. Appl. Genet. 128, 353–363. doi: 10.1007/s00122-014-2435-3 25490985

[B83] Suprayogi Y. Pozniak C. J. Clarke F. R. Clarke J. M. Knox R. E. Singh A. K. (2009). Identification and validation of quantitative trait loci for grain protein concentration in adapted Canadian durum wheat populations. Theor. Appl. Genet. 119, 437–448. doi: 10.1007/s00122-009-1050-1 19462147

[B84] Thorwarth P. Ahlemeyer J. Bochard A. M. Krumnacker K. Blümel H. Laubach E.. (2017). Genomic prediction ability for yield-related traits in German winter barley elite material. Theor. Appl. Genet. 130, 1669–1683. doi: 10.1007/s00122-017-2917-1 28534096

[B85] Tian X. Engel B. A. Qian H. Hua E. Sun S. Wang Y. (2021). Will reaching the maximum achievable yield potential meet future global food demand? J. Cleaner Prod. 294, 126285. doi: 10.1016/j.jclepro.2021.126285

[B86] Tsenov N. Gubatov T. Yanchev I. (2020). Correlations between grain yield and related traits in winter wheat under multi-environmental traits. Agric. Sci. Technol. 12, 295–300. doi: 10.15547/issn1314-412X

[B87] Uauy C. Distelfeld A. Fahima T. Blechl A. Dubcovsky J. (2006). A NAC gene regulating senescence improves grain protein, zinc, and iron content in wheat. Science 314, 1298–1301. doi: 10.1126/science.1133649 17124321 PMC4737439

[B88] van der Biezen E. A. Jones J. D. (1998). The NB-ARC domain: a novel signalling motif shared by plant resistance gene products and regulators of cell death in animals. Curr. Biol. CB 8 (7), R226–R227. doi: 10.1016/s0960-9822(98)70145-9 9545207

[B89] van Ittersum M. K. van Bussel L. G. J. Wolf J. Grassini P. van Wart J. Guilpart N.. (2016). Can sub-Saharan Africa feed itself? Proc. Natl. Acad. Sci. U. S. A. 113, 14964–14969. doi: 10.1073/pnas.1610359113 27956604 PMC5206509

[B90] Vishwakarma M. K. Arun B. Mishra V. K. Yadav P. S. Kumar H. Joshi A. K. (2016). Marker-assisted improvement of grain protein content and grain weight in Indian bread wheat. Euphytica 208, 313–321. doi: 10.1007/s10681-015-1598-6

[B91] Vishwakarma M. K. Mishra V. K. Gupta P. K. Yadav P. S. Kumar H. Joshi A. K. (2014). Introgression of the high grain protein gene Gpc-B1 in an elite wheat variety of Indo-Gangetic Plains through marker assisted backcross breeding. Curr. Plant Biol. 1, 60–67. doi: 10.1016/j.cpb.2014.09.003

[B92] Weis C. Hückelhoven R. Eichmann R. (2013). LIFEGUARD proteins support plant colonization by biotrophic powdery mildew fungi. J. Exp. Bot. 64 (12), 3855–3867. doi: 10.1093/jxb/ert217 23888068 PMC3745739

[B93] Woese C. R. Olsen G. J. Ibba M. Söll D. (2000). Aminoacyl-tRNA synthetases, the genetic code, and the evolutionary process. Microbiol. Mol. Biol. Rev. MMBR 64 (1), 202–236. doi: 10.1128/MMBR.64.1.202-236.2000 10704480 PMC98992

[B94] Woo J. S. Imm J. H. Min C. K. Kim K. J. Cha S. S. Oh B. H. (2006). Structural and functional insights into the B30.2/SPRY domain. EMBO J. 25 (6), 1353–1363. doi: 10.1038/sj.emboj.7600994 16498413 PMC1422157

[B95] Worland A. J. (1996). The influence of flowering time genes on environmental adaptability in European wheats. Euphytica 89, 49–57. doi: 10.1007/BF00015718

[B96] Würschum T. Leiser W. L. Langer S. M. Tucker M. R. Longin C. F.H. (2018). Phenotypic and genetic analysis of spike and kernel characteristics in wheat reveals long-term genetic trends of grain yield components. Theor. Appl. Genet. 131, 2071–2084. doi: 10.1007/s00122-018-3133-3 29959471

[B97] Xu X. Bai G. Carver B. F. Shaner G. E. (2005). A QTL for early heading in wheat cultivar Suwon 92. Euphytica 146, 233–237. doi: 10.1007/s10681-005-9017-z

[B98] Yang Y. Chai Y. Zhang X. Lu S. Zhao Z Z. Wei D. (2020). Multi-locus GWAS of quality traits in bread wheat: mining more candidate genes and possible regulatory network. Front. Plant Sci. 11. doi: 10.3389/fpls.2020.01091 PMC7411135 32849679

[B99] Yu J. Pressoir G. Briggs W. H. Vroh Bi I. Yamasaki M. Doebley J. F.. (2006). A unified mixed-model method for association mapping that accounts for multiple levels of relatedness. Nat. Genet. 38, 203–208. doi: 10.1038/ng1702 16380716

[B100] Zampieri M. Ceglar A. Dentener F. Toreti A. (2017). Wheat yield loss attributable to heat waves, drought and water excess at the global, national and subnational scales. Environ. Res. Lett. 12, 064008. doi: 10.1088/1748-9326/aa723b

[B101] Zanke C. Ling J. Plieske J. Kollers S. Ebmeyer E. Korzun V.. (2014). Genetic architecture of main effect QTL for heading date in European winter wheat. Front. Plant Sci. 5. doi: 10.3389/fpls.2014.00217 PMC4033046 24904613

[B102] Zhang P. Guo. G. Wu. Q. Chen Y. Xie J. Lu P.. (2020). Identification and fine mapping of spot blotch (Bipolaris sorokiniana) resistance gene Sb4 in wheat. Theor. Appl. Genet. 133, 2451–2459. doi: 10.1007/s00122-020-03610-3 32451599

[B103] Zhang K. Tian J. Zhao L. Liu B. Chen G. (2009). Detection of quantitative trait loci for heading date based on the doubled haploid progeny of two elite Chinese wheat cultivars. Genetica 135, 257–265. doi: 10.1007/s10709-008-9274-6 18500653

[B104] Zhang C. Zheng B. He Y. (2021). Improving grain yield via promotion of kernel weight in high yielding winter wheat genotypes. Biology 11, 42. doi: 10.3390/biology11010042 35053040 PMC8772892

[B105] Zhu C. Gore M. Buckler E. S. Yu J. (2008). Status and prospects of association mapping in plants. Plant Genome 1, 1. doi: 10.3835/plantgenome2008.02.0089

