# Supplementary figures and images for: Genetic dissection of value-added quality traits and agronomic parameters through genome-wide association mapping in bread wheat (T. aestivum L.)
Source: Front Plant Sci. 2024 Aug 20;15:1419227. doi: 10.3389/fpls.2024.1419227 (PMC11368860; doi:10.3389/fpls.2024.1419227)

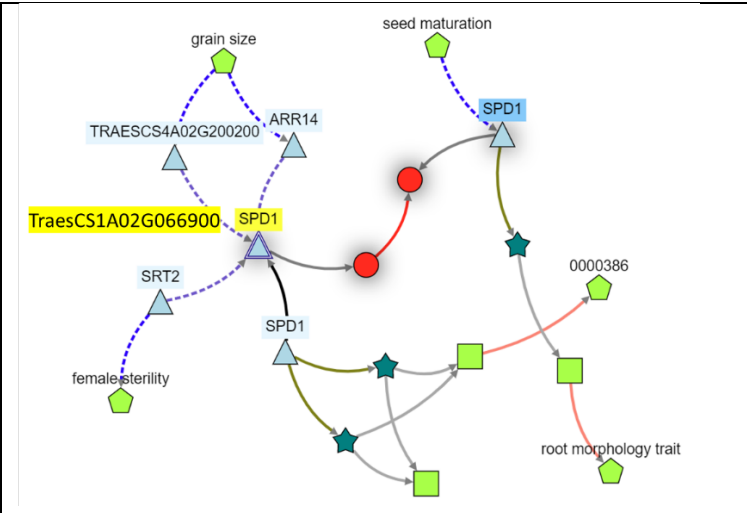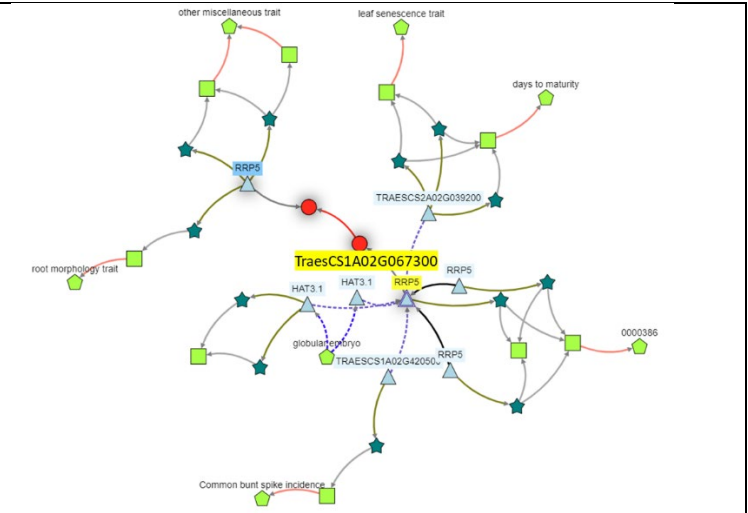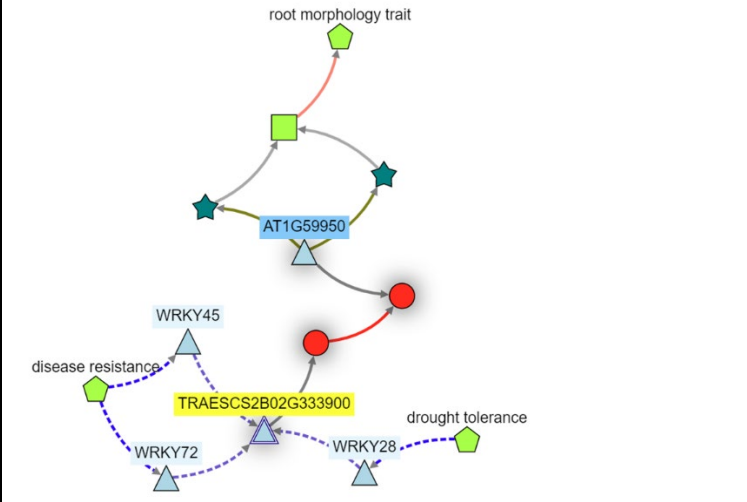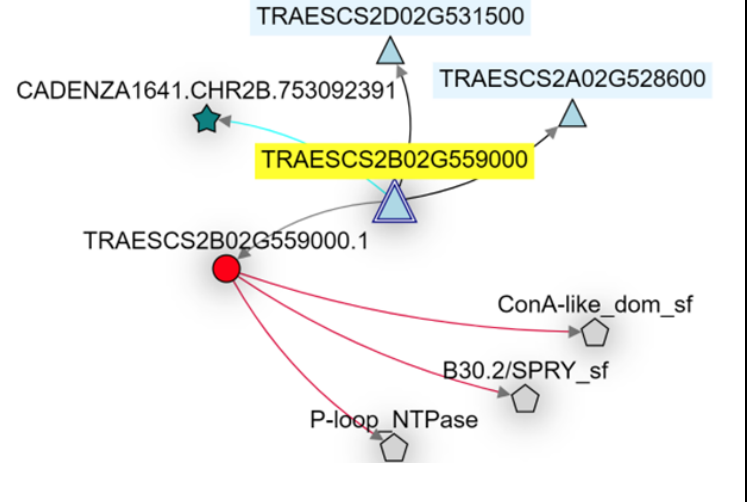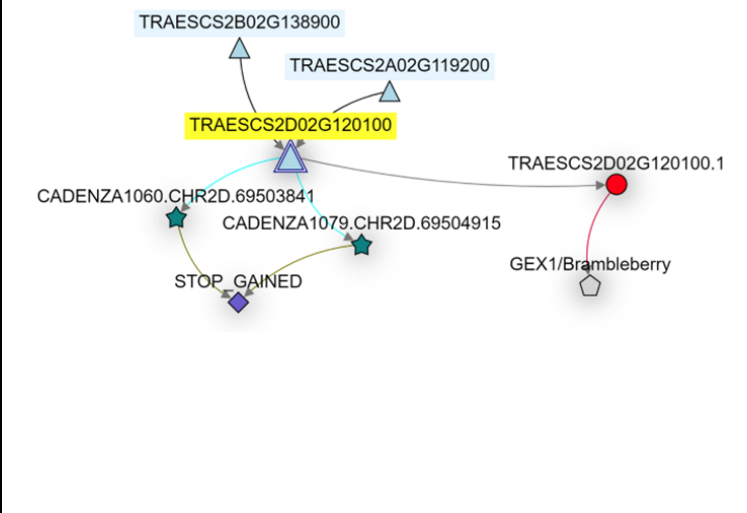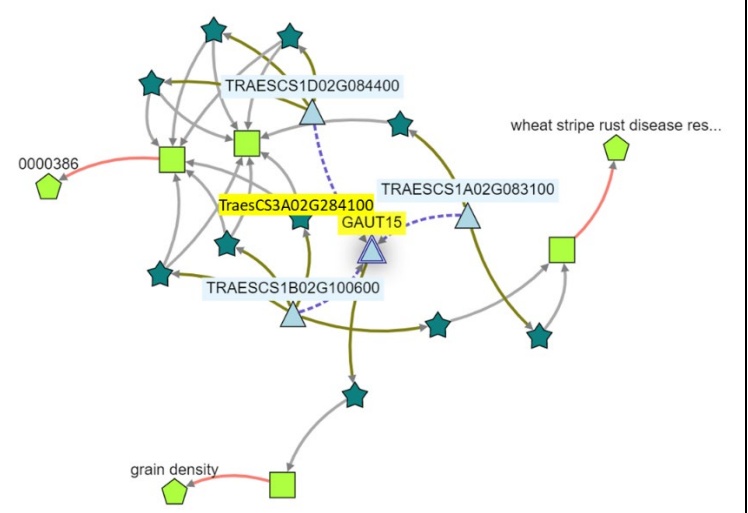



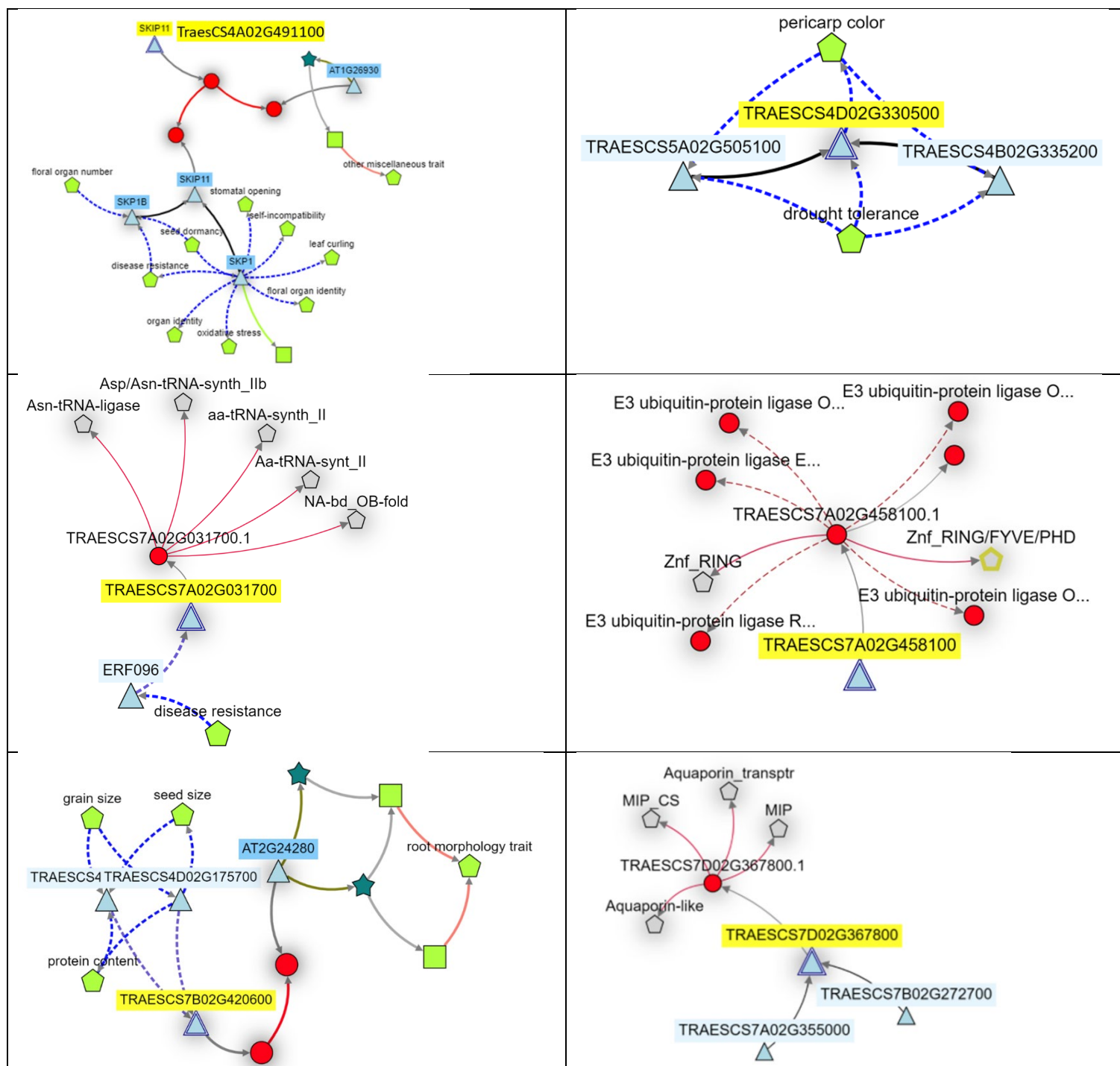

Supplementary Figure 1: Visualization of gene networks using Knetminer

Supplement: Supplementary file 1 [file Image1.pdf]
